# Translated Long Non-Coding Ribonucleic Acid ZFAS1 Promotes Cancer Cell Migration by Elevating Reactive Oxygen Species Production in Hepatocellular Carcinoma

**DOI:** 10.3389/fgene.2019.01111

**Published:** 2019-11-12

**Authors:** Zhi-Wei Guo, Yu Meng, Xiang-Ming Zhai, Chen Xie, Na Zhao, Min Li, Chun-Lian Zhou, Kun Li, Tian-Cai Liu, Xue-Xi Yang, Ying-Song Wu

**Affiliations:** ^1^School of Laboratory Medicine and Biotechnology, Institute of Antibody Engineering, Southern Medical University, Guangzhou, China; ^2^Key Laboratory of Gene Engineering of the Ministry of Education, School of Life Sciences, Sun Yat-sen University, Guangzhou, China; ^3^Department of Molecular and Cellular Biology, Baylor College of Medicine, Houston, TX, United States

**Keywords:** hepatocellular carcinoma, translated small open reading frames, ribosome-protected fragment sequencing, ZFAS1, reactive oxygen species

## Abstract

Micropeptides (≤100 amino acids) are essential regulators of physiological and pathological processes, which can be encoded by small open reading frames (smORFs) derived from long non-coding RNAs (lncRNAs). Recently, lncRNA-encoded micropeptides have been shown to have essential roles in tumorigenesis. Since translated smORF identification remains technically challenging, little is known of their pathological functions in cancer. Therefore, we created classifiers to identify translated smORFs derived from lncRNAs based on ribosome-protected fragment sequencing and machine learning methods. In total, 537 putative translated smORFs were identified and the coding potential of five smORFs was experimentally validated *via* green fluorescent protein-tagged protein generation and mass spectrometry. After analyzing 11 lncRNA expression profiles of seven cancer types, we identified one validated translated lncRNA, ZFAS1, which was significantly up-regulated in hepatocellular carcinoma (HCC). Functional studies revealed that ZFAS1 can promote cancer cell migration by elevating intracellular reactive oxygen species production by inhibiting nicotinamide adenine dinucleotide dehydrogenase expression, indicating that translated ZFAS1 may be an essential oncogene in the progression of HCC. In this study, we systematically identified translated smORFs derived from lncRNAs and explored their potential pathological functions in cancer to improve our comprehensive understanding of the building blocks of living systems

## Introduction

Hepatocellular carcinoma (HCC) accounts for more than 90% of primary liver cancers and is the sixth most common malignancy worldwide. Moreover, it is the third leading cause of cancer death. Despite intensive investigations and therapeutic improvements, the 5-year overall survival rate for HCC is merely 18% (Siegel et al., 2019), highlighting the urgent need to clarify novel mechanisms contributing to liver malignancy.

Recent genome-wide studies have revealed that small open reading frames (smORFs) concealed in long non-coding RNAs (lncRNAs) could encode micropeptides (≤100 amino acids) with essential roles in the regulation of physiological and pathological processes of various species (Guttman et al., 2013; Magny et al., 2013; Bazzini et al., 2014; Pauli et al., 2014; Anderson et al., 2015; Calviello et al., 2016). For example, in *Drosophila*, the lncRNA pncr003:2L encodes two micropeptides that regulate cardiac contraction (Magny et al., 2013). Meanwhile, in zebrafish, a micropeptide called Toddler can activate the extracellular-signal-regulated kinase pathway to promote embryogenesis (Pauli et al., 2014). Moreover, in human, the lncRNA-encoded micropeptide myoregulin (MLN) is an important regulator of skeletal muscle performance that directly inhibits the sarco/endoplasmic reticulum calcium-ATPase to control muscle relaxation by regulating calcium ion uptake into the sarcoplasmic reticulum (Anderson et al., 2015). More importantly, micropeptides encoded by lncRNAs have been demonstrated to have essential roles in tumorigenesis. For example, the lncRNA HOXB-AS3 encodes a 53 amino acid micropeptide that affects clone cell metabolism to suppress cancer progression by competitively binding with the RNA binding protein *hnRNP A1* to inhibit the splicing of pyruvate kinase (Huang et al., 2017). Owing to their critical functions, it is necessary to systematically identify translated smORFs derived from lncRNAs and explore their potential physiological and pathological functions to comprehensively elucidate the building blocks of living systems.

Precise identification of translated smORFs derived from lncRNAs is prerequisite of their functional studies (Kong et al., 2007; Olexiouk et al., 2016; Xiao et al., 2018). However, evaluating the protein-coding potential of smORFs remains challenging for conventional prediction methods. Meanwhile, traditional translated ORF prediction mainly relies on the ORF size, sequence evolutionary conservation, and mass spectrometry (MS) data. However, the features of smORFs and translated ORFs of protein-coding genes differ substantially. Because the majority of smORFs are derived from lncRNAs, their expression levels and conservation scores are generally lower than ORFs of protein-coding genes. Moreover, they are considerably shorter than 300 nucleotide (nt) in length, which is typically used as a filter parameter in prediction methods to reduce the false positive rate before model construction. Therefore, novel methods are urgently needed to identify translated smORFs from the vast number of untranslatable smORFs.

Recent advances in high-throughput sequencing of ribosome-protected mRNA fragments (RPF-Seq) have enabled systematic identification of transcripts combined with ribosomes. Ribosome features of coding and non-coding ORFs quantified by RPF-Seq exhibit significant differences, which could be applied to identify translated smORFs (Guttman et al., 2013; Bazzini et al., 2014). Because translated ORFs must bind to ribosomes for protein translation, smORFs that do not bind to ribosomes can first be filtered out. However, since non-coding ORFs can also bind to ribosomes, additional ribosome features are required to identify translated smORFs, such as ribosome footprinting and ribosome release. Ribosome footprinting separates coding ORFs from non-coding ORFs according to the unbalanced distribution of RPF-Seq in the reading frame (Bazzini et al., 2014). Besides, ribosomes are released when they meet stop codons; therefore, a disequilibrium in the number of ribosomes on each side of stop codons could be assessed to determine the coding potential of smORFs (Guttman et al., 2013). However, these features lack effective integration in systematic assessments of the coding potential of smORFs derived from lncRNAs.

Herein, we first predicted translated smORFs using newly developed classifiers based on three ribosome features derived from two RPF-Seq datasets and four machine-learning models. To further investigate their pathological functions in cancer, we determined their composition and abundance in seven cancer types by analyzing 11 lncRNA microarray datasets. Finally, we found one validated translated lncRNA ZFAS1, which promoted HCC cell migration and explored the underlying mechanisms. In summary, this study identified hundreds of translated smORFs and was trying to reveal their roles in cancer pathogenesis.

## Methods

### Definition of Coding and Non-Coding Open Reading Frames

Reference transcripts of protein-coding genes were downloaded from UCSC RefSeq (Casper et al., 2018). Each stop codon (UAA, UAG, or UGG) paired with the most distal in-frame AUG start codon without an intervening stop was defined as an ORF. In cases where one gene corresponded to multiple transcripts, the longest was retained. Translated ORFs of protein-coding genes were annotated with RefSeq and collated as the positive dataset. The negative dataset of translated ORFs consisted of ORFs derived from the 5′ and 3′ untranslated regions (UTRs). Based on the literature, upstream and downstream ORFs in the 5′ and 3′ UTRs with protein-coding potential were filtered (Vilela and Mccarthy, 2003; Oyama et al., 2004; Fritsch et al., 2012; Chew et al., 2013; Guttman et al., 2013; Slavoff et al., 2013; Bazzini et al., 2014; Calviello et al., 2016). To identify translated lncRNAs, lncRNA sequences were downloaded from Ensembl (Zerbino et al., 2018) and GENCODE (Harrow et al., 2012), and all smORFs shorter than 350 nt in length were identified.

### Sequencing of Ribosome-Protected Messenger Ribonucleic Acid Fragment Data Analysis

Human RPF-Seq datasets of U2OS and HeLa cells (GSE61073 and GSE21992) were obtained from National Center for Biotechnology Information Gene Expression Omnibus (NCBI GEO) (Guo et al., 2010; Eichhorn et al., 2014). The adaptor sequences of each read were removed, and reads aligned to translation-related RNAs [ribosomal RNA, transfer RNA, mitochondrial RNA, and mitochondrial ribosomal RNA] were filtered. The remaining reads were aligned to the human reference genome (hg19) using Bowtie (ver. 0.12.9) with 27–32 nt (Langmead et al., 2009). Unmapped reads were realigned to the reference transcripts to capture reads spanning two exons. The genomic position of 13^th^ nucleotide of the mapped read was regarded as its position.

### Translated Small Open Reading Frame Prediction

**Figure 1:**
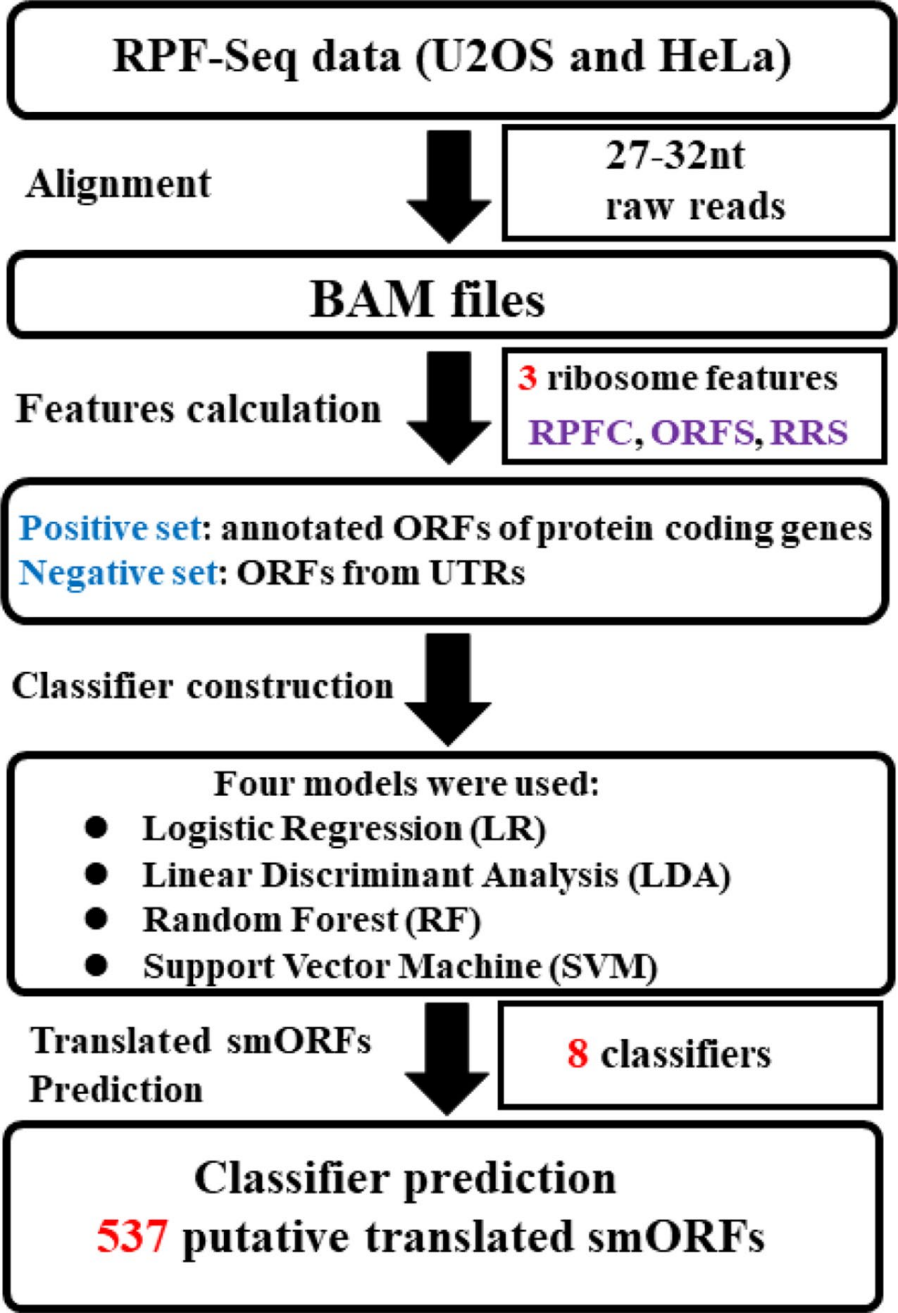
Systematic overview of translated small open reading frame (smORF) prediction. Two sequencing of ribosome-protected mRNA fragments (RPF-Seq) originated from U2OS and HeLa cells were applied to respectively calculate three ribosome features of the positive and negative datasets. Three features of each RPF-Seq combined with one of machine learning models could create one classifier. As four classification models were used, four classifiers were developed to predict the translated smORFs in each RPF-Seq dataset. RPFC, ribosome-protected mRNA fragments coverage; ORFS, ORF score; RRS, ribosome release score.

**Figure 1** presents an overview of the translated smORFs prediction. Briefly, three ribosome features of positive and negative datasets were calculated respectively from the U2OS and HeLa RPF-Seq: ORF score (ORFS) (Bazzini et al., 2014), ribosome release score (RRS) (Guttman et al., 2013), and RPF coverage (RPFC) (Bazzini et al., 2014). The ORFs in the positive and negative datasets with three feature values simultaneously equal to zero were filtered. The remaining ORFs in the positive and negative datasets were split into training and validation cohort (70% training and 30% validation, **Supplementary Table S1**). Combined with these three ribosome features, four machine-learning models, including random forest (RF) (Breiman, 2001), logistic regression (LR), linear discriminant analysis (LDA), and support vector machine (SVM) models, were employed to construct the prediction classifiers. For SVM, we used the e1071 package of R software with the non-linear kernel. For RF, we used the randomForest package of R software with the default setting. The predictive accuracy of the classifiers was estimated *via* leave-one-out cross validation. Receiver operating characteristic (ROC) curves were plotted using R (ver. 3.3.1), and the pROC package (Robin et al., 2011) was applied to assess differences in the area under the ROC curve (AUC). Finally, the classifiers were applied to identify translated smORFs derived from lncRNAs.

The ORFS was calculated as:

$$\text{ORFS}=\log_{2}\left(\left(\overset{3}{\underset{i\;=\;1}{\sum }}\frac{{\left(C_{i}-\bar{C}\right)}^{2}}{\bar{C}}\right)+1\right)$$

$$\times \{\begin{array}{c} -1,\;if\;(C_{1}<C_{2})\cup (C_{1}<C_{3})\\ 1,\;other \end{array}$$

where C_i_ is the number of reads in reading frame i and $\bar{\text{C}}$ the mean number of reads in the reading frame.

RRS was calculated as:

$$\text{RRS}=\log_{2}\left(\left(\overset{2}{\underset{i\;=1}{\sum }}\frac{{\left(C_{i}-\bar{C}\right)}^{2}}{\bar{C}}\right)+1\right)$$

$$\times \{\begin{array}{c} -1,\;if\;(C_{1}\leq C_{2})\\ 1,\;other \end{array}$$

For annotated protein-coding genes, C_1_ and C_2_ are the number of reads in translated ORFs and 3′ UTRs, respectively. For non-coding transcripts, C_1_ is the number of reads in ORFs and C_2_ is the number of reads in the regions ranging from the stop codon to the next start codon. $\bar{\text{C}}$ the mean number of reads for C_1_ and C_2_.

RPFC was calculated as:

$$\text{RPFC}=C_{i}/C_{m}$$

where $C_{i}$ the number of $1^{st}$ reading frames covered by RPF-Seq and $C_{m}$ the number of $1^{st}\;$ reading frame in the ORF.

### Translated Small Open Reading Frame Validation

We applied two experimental methods to validate the protein-coding potential of the smORFs: MS and construct generation. To detect small proteins, unfractionated samples and small protein-enriched fractions were prepared from HeLa cells. Proteins less than 15 kDa were excised, and then treated and detected using a protocol similar to that of a previous study (Bazzini et al., 2014). MS data was analyzed using the ANDROMEDA search algorithms in MaxQuant (ver. 1.4.0.5) at a false discovery rate (FDR) of 0.05 (Cox et al., 2011). Peptide fragments mapped to lncRNA-encoded micropeptides were further aligned to the NCBI non-redundant protein sequence database to filter false positive results using BLAST. For construct generation, five translated lncRNAs were selected (ENST00000458653, ENST00000586949, ENST00000602483, ENST00000444717, and ENST00000417112) according to the prediction results of different classifiers. Then a series of vectors were generated in which the 5′ UTR-ORFs in the full-length transcripts were fused to a GFP with a mutation (GFPmut) in which the green fluorescent protein (GFP) start codon ATGGTG was mutated to ATTGTT (**Supplementary Table S2** and **Supplementary Figure S4**). In addition, the vectors of positive and negative controls, including 5′UTR-ORF-GFPmut (GAPDH) and GFPmut were generated (**Supplementary Figure S1**).

### Expression Profiles of ZFAS1 in Multiple Tumor and Normal Tissues

Eleven cancer-related lncRNA expression profiles measured with the Arraystar LncRNA Microarray V2.0 platform were retrieved from NCBI GEO (**Supplementary Table S3)** (Yan et al., 2013; Chen et al., 2015; Gu et al., 2015; Kim et al., 2015; Zhang et al., 2015; Liao et al., 2016; Qin et al., 2016; Cao et al., 2017). The lncRNA expression profiles were extracted and normalized using GEOquery (Davis and Meltzer, 2007). Then, paired Wilcoxon rank sum test was used to identify significantly differentially expressed lncRNAs. The P-value was adjusted to the FDR using the Benjamini–Hochberg procedure. An FDR ≤ 0.1 and |log_2_ fold change| ≥ 0.6 were considered as criteria of significantly dysregulated lncRNAs. ZFAS1 expression levels of 25 normal tissues were downloaded from the The Genotype-Tissue Expression (GTEx), 2013. Reverse transcription quantitative polymerase chain reaction (RT-qPCR) was used to validate the ZFAS1 expression change in HCC cells. The total RNA of 32 pairs of HCC tissues and matching adjacent normal tissues was isolated with TRI reagent (Cat. T9424, Sigma) following the manufacturer’s instructions. Reverse transcription was performed with total RNA using Maxima H Minus First Strand cDNA Synthesis Kit (Cat. K1652, Thermo Fisher Scientific, Waltham, MA). QpCR analysis was performed on Eppendorf RealPlex using SYBR FAST qPCR Kits (Cat. KK4602, Kapa Biosystems, Wilmington, MA). All reactions were run in triplicates. The relative expression levels of target genes were normalized to the expression of internal control genes, GAPDH, which yielded 2^−ΔΔCt^ values. RT-qPCR primers were as follows: ZFAS1 forward, 5′-GCGGGTACAGAATGGATTTTGG-3′ and reverse, 5′-CAACACCCGCATTCATCCTG-3′; GAPDH forward: 5′-GAGTCAACGGATTTGGTCGT-3′ and reverse, 5′-GACAAGCTTCCCGTTCTCAG-3′. Kolmogorov-Smimov test (K-S test) was used to test whether the data was normally distributed. Brown-Forsythe test was used to test whether the variance was equal.

### Knockdown and Overexpression of Translated Small Open Reading Frames of ZFAS1

Two small interfering RNA (siRNA) oligonucleotides were designed and synthesized for RNA interference knockdown. The guide strands of two siRNAs were as follows: 5′-CCAAGGAAGCCACGUGCAG-3′ and 5′-AUACAUAGCCUGAGUUUAA-3′. SK-Hep1 cells were transfected with siRNA oligonucleotides at a final concentration of 50 nM using Lipofectamine 2000 Reagent (Life Technologies), according to the manufacturer’s instructions. To assess overexpression of the translated lncRNA ZFAS1, the cDNA of ZFAS1 translated smORF was amplified and subcloned into the BamHI and EcoRI sites of pcDNA3.0 expression vector. Then ZFAS1 expression level of SK-Hep1 transfected with siRNAs or plasmids was detected by RT-qPCR.

### Sequencing of Ribosome-Protected Messenger Ribonucleic Acid Fragment and Data Analysis

Total RNA was extracted from ZFAS1-overexpression and RNA interference SK-Hep1 cells using a High Purity RNA Isolation Kit (Thermo Fisher Scientific, Waltham, MA) with two biological replicates. Library preparation and sequencing were performed using Ion Proton at DaRui Biotechnology Corporation (Guangzhou, China). The sequencing data are available on GEO (GSE104226). The data were analyzed using the Torrent Suite and default RNA-Seq analysis plug-in (life technology) to generate normalized gene expression profiles. Differentially expressed genes were identified using edgeR with their raw count (Robinson et al., 2010) and the *P-value* was adjusted to the FDR. An FDR ≤ 0.1 and |log_2_ fold change| ≥ 0.6 were considered as criteria of significantly dysregulated genes, and functional enrichment analysis was performed to analyze enriched gene ontology (GO) terms using clusterProfiler (Yu et al., 2012).

### Cell Motility and Reactive Oxygen Species Level Detection

Cell motility was evaluated with a transwell assay. SK-Hep1 cells transfected with siRNAs and expression plasmids were cultured in the upper chamber of transwell plates for 48 h. The membranes were stained with crystal violet and the migration of cells was photographed and measured with an ELISA Microplate Reader, with five replicates (Bio-Rad, Hercules, CA, USA). For reactive oxygen species (ROS) detection, MitoSOX superoxide (M36008, Molecular Probes™ Invitrogen Detection Technologies) was dissolved in dimethyl sulfoxide. The cell culture medium was removed, washed twice with phosphate-buffered saline (PBS), and MitoSOX reagent was added and incubated for 10 min. Next, the MitoSOX reagent was removed and cells were collected and washed twice with PBS. The average fluorescence intensity of the cells was observed with flow cytometry. The gene expression correlation between superoxide dismutase 2 (SOD2) and oxidative phosphorylation-related genes was evaluated with the Pearson correlation coefficient using ready-analyzed gene expression profiles of HCC patients derived from The Cancer Genome Atlas (TCGA).

## Results

### Identification of 537 Putative Coding Small Open Reading Frames

**Figure 2:**
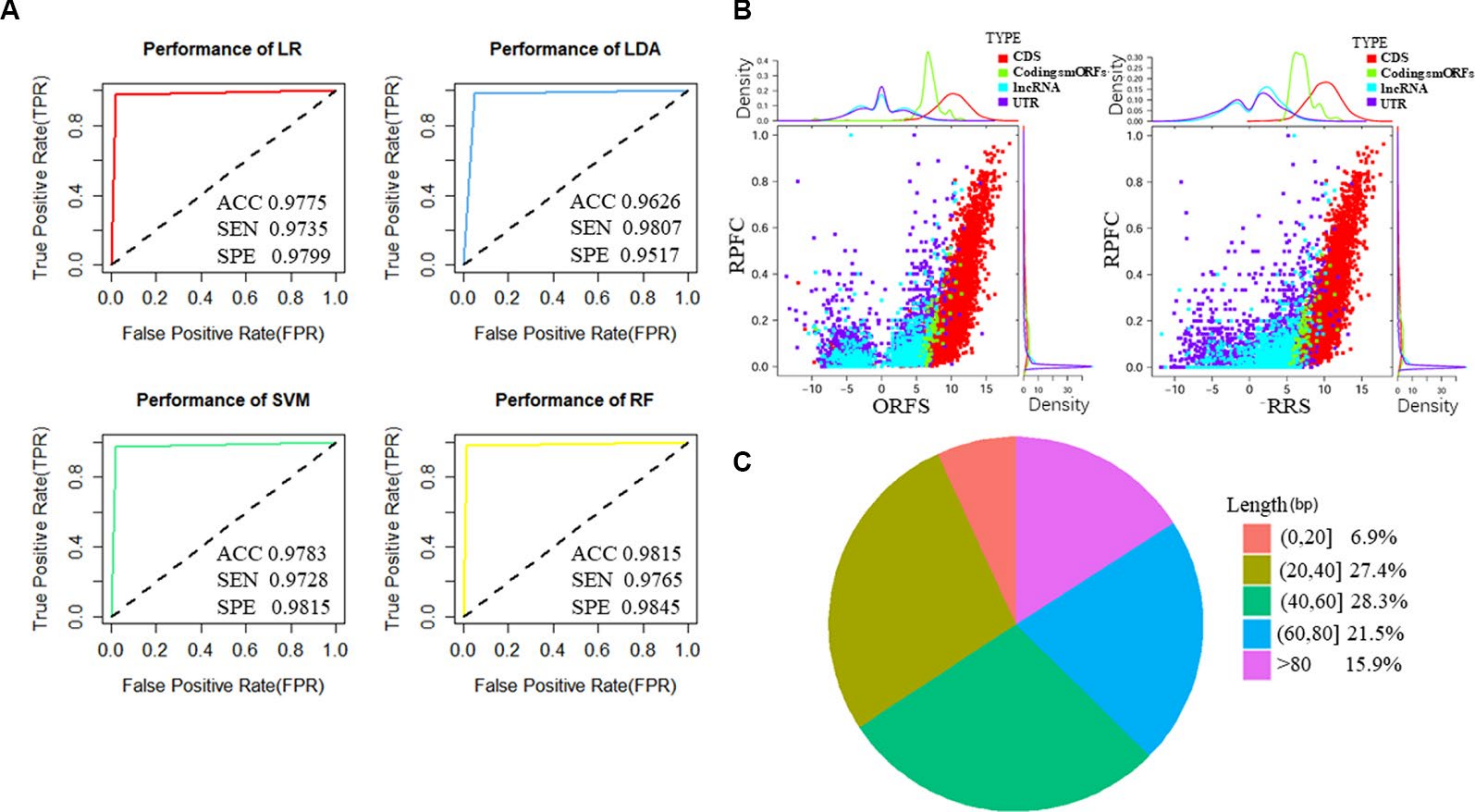
Features of predicted translated small open reading frames (smORFs) based on U2OS sequencing of ribosome-protected mRNA fragments (RPF-Seq). **(A)** The performance of the four classifiers based on logistic regression (LR), linear discriminant analysis (LDA), support vector machine (SVM), and random forest models (RF). **(B)** Ribosome features of different ORFs. The ribosome release score and ORF score values of putative translated smORFs were similar, and were higher than ORFs derived from untranslated regions (UTRs) and long non-coding RNAs (lncRNAs) but lower than annotated ORFs of protein-coding genes. The ribosome-protected mRNA fragment coverage scores of translated smORFs and protein-coding genes were similar, but their distributions differed substantially from UTRs and lncRNAs. **(C)** Length distribution of micropeptides encoded by putative translated smORFs. ACC, accuracy; SEN, sensitivity; SPE, specificity; RPFC, ribosome-protected mRNA fragments coverage; ORFS, ORF score; RRS ribosome release score.

To train the classifier models, translated ORFs of protein-coding genes and non-translated ORFs with ribosome combination were used as positive and negative controls, respectively. Three ribosome features of all smORFs were quantified to evaluate their protein-coding potential: the RPFC reflected the number of ribosomes combined with ORFs (Bazzini et al., 2014), and the RRS and ORFS reflected whether the transcripts were translated (Guttman et al., 2013; Bazzini et al., 2014). Compared with the negative controls, the values of three ribosome features were significantly raised in the translated ORF dataset (all *P-value* < 0.0001, Student’s *t*-test, **Supplementary Figure S2**). Four classification models were applied to construct classifiers, including LR, LDA, SVM, and RF with three ribosome features. We applied ROC curve to present the AUC, accuracy, sensitivity, and specificity of the classifiers. All classifiers had AUCs higher than 0.947 in the training cohort (**Table 1**, **Figure 2A**, and **Supplementary Figure S3A**). The RF model were observed with the highest AUC values (0.998) among the four classifiers in U2OS RPF-Seq while the LDA model with the lowest AUC values (0.966, **Table 1**). By contrast with the training cohort, their AUC was similar to their performance in the internal validation cohort except for the classifiers based on RF models (**Table 1**).

**Table 1:** Performance of classifiers.

Model	Training cohort					Validation Cohort			*P*
	AUC (95% CI)	Acc (%)	Sen (%)	Spe (%)	AUC (95% CI)	Acc (%)	Sen (%)	Spe (%)	
LR_U	0.977 (0.973–0.980)	97.75	97.35	97.99	0.977 (0.972–0.983)	97.80	97.51	97.97	0.83
LDA_U	0.966 (0.962–0.970)	96.26	98.07	95.17	0.963 (0.957–0.969)	95.83	98.07	94.48	0.36
SVM_U	0.977 (0.974–0.981)	97.83	97.28	98.15	0.977 (0.971–0.982)	97.77	97.27	98.06	0.88
RF_U	0.998 (0.997–0.999)	98.15	97.65	98.45	0.976 (0.971–0.982)	97.83	97.34	98.12	3e−14
LR_H	0.961 (0.956–0.966)	96.30	95.07	97.17	0.965 (0.958–0.972)	96.60	95.97	97.06	0.36
LDA_H	0.947 (0.941–0.952)	94.68	94.49	94.81	0.938 (0.929–0.947)	93.66	94.37	93.16	0.10
SVM_H	0.961 (0.957–0.966)	96.36	94.96	97.35	0.965 (0.958–0.97)	96.64	95.71	97.30	0.42
RF_H	0.998 (0.998–0.999)	97.81	97.17	98.27	0.976 (0.970–0.981)	97.58	96.67	98.25	1e−14

LR_U, LDA_U, SVM_U, and RF_U means the classifiers based on ribosome-protected fragment sequencing of U2OS cells using logistic regression, linear discriminant analysis, support vector machine, and random forest models, respectively. LR_H, LDA_H, SVM_H, and RF_H means the classifiers based on ribosome-protected fragment sequencing of HeLa cells using logistic regression, linear discriminant analysis, support vector machine, and random forest models, respectively. AUC, the area under ROC curve; Acc, accuracy; Sen, sensitivity; Spe, specificity; P. P value of the AUC difference between training and validation cohort.

To identify novel translated smORFs derived from lncRNAs, we applied the classifiers to assess the coding potential of smORFs derived from previously annotated lncRNAs and uncharacterized processed transcripts from Ensembl. To identify as many micropeptides as possible, we took the union of the classifiers based on different models and we identified 537 putative translated smORFs concealed in 463 lncRNA transcripts (**Supplementary Table S4**). The RRS and ORFS values of the putative coding smORFs were similar, and were higher than the ORFs derived from UTRs and lncRNAs, but lower than the annotated ORFs of protein-coding genes (**Figure 2B** and **Supplementary Figure S2B**). The RPFC scores of translated smORFs and protein-coding genes were similar, but their distribution differed substantially from UTRs and lncRNAs (**Figure 2B** and **Supplementary Figure S2B**). Moreover, most of the micropeptides were shorter than 80 amino acids, which accounted for approximately 84% of putative coding smORFs (**Figure 2C**).

### Experimental Validation of Five Coding Small Open Reading Frames

**Figure 3:**
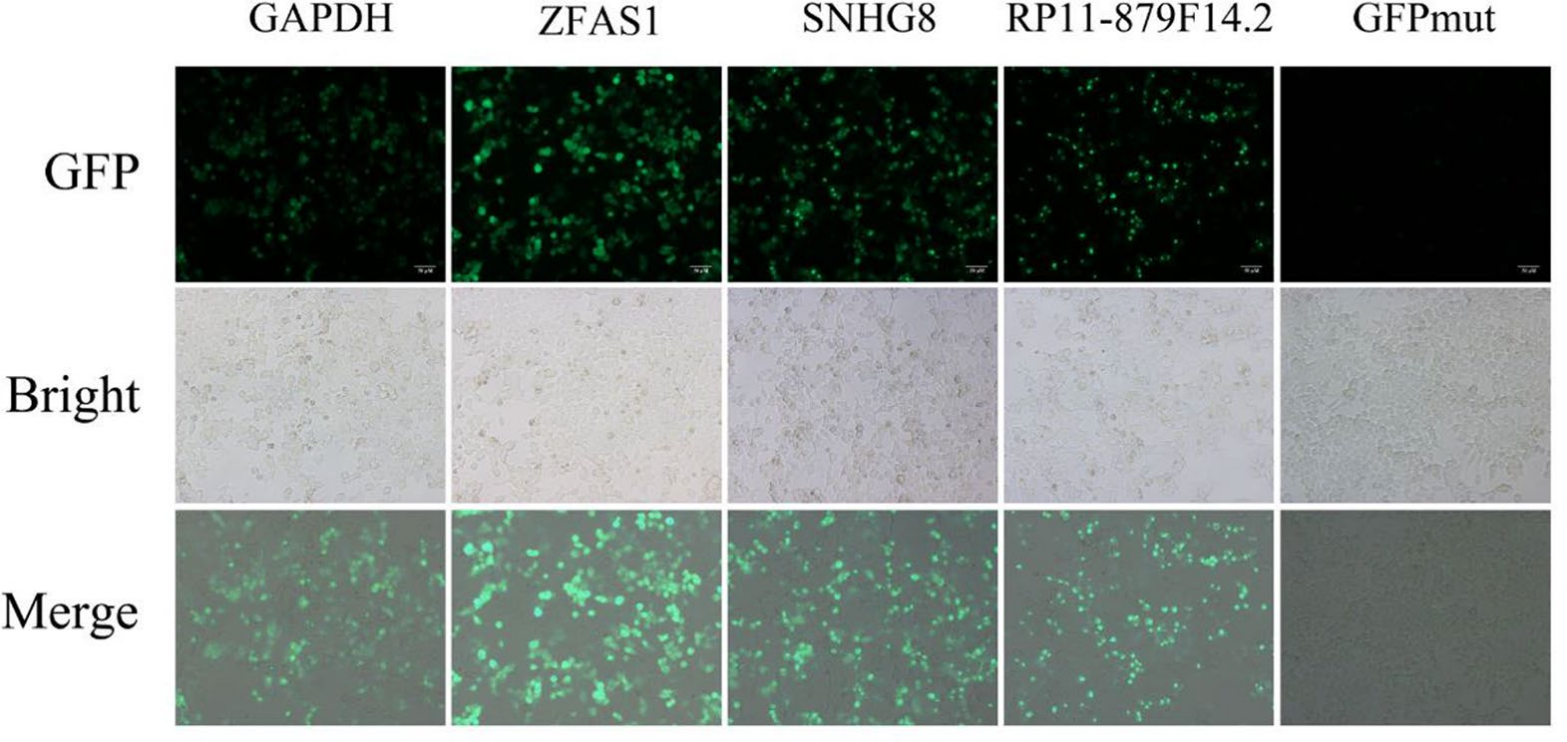
Constructs generated to validate the protein-coding potential of small open reading frames. The 5′UTR-ORFs of glyceraldehyde 3-phosphate dehydrogenase, ZFAS1, SNHG8, and RP11-879F14.2 were fused to a GFPmut in which the green fluorescent protein start codon ATGGTG was mutated to ATTGTT. Substantial amounts of fusion protein were detected following transfection of the constructs into SK-Hep1 cells. In addition, the fusion protein was abolished after SK-Hep1 cells were transfected with the GFPmut construct.

Five putative translated lncRNAs were chosen to validate their protein-coding potential according to the number of four classifiers by which they were predicted (**Supplementary Tables S4** and **S5**). Three translated smORFs (ZFAS1, RP11-879F14.2, SNHG8) were simultaneously predicted by four machine learning models in the U2OS RPF-Seq and two translated smORFs (RP4-614O4.11 and RP11-554I8.2) were predicted by only one of the models. Then, a series of vectors were generated, including 5′UTR-ORF-GFPmut (GAPDH, ZFAS1, RP11-879F14.2, SNHG8, RP4-614O4.11, and RP11-554I8.2) and GFPmut (**Supplementary Figure S1**). After transfecting SK-Hep1 cells with these constructs, substantial fusion protein expression was observed in ZFAS1-, RP11-879F14.2-, and SNHG8-transfected cells, while cancer cells transfected with GFPmut abolished fusion protein expression (**Figure 3**), indicative of their coding potential. However, the fusion protein was not translated in SK-Hep1 cells transfected with the vectors of RP4-614O4.11 and RP11-554I8.2 constructs, indicating that they did not possess coding potential (data not shown). In addition, the peptide fragments of two putative lncRNA-encoded micropeptides were detected by MS (**Supplementary Table S6**). These peptides contained 100 and 112 amino acids, and were derived from RP11-277P12.20 and LINC00909, respectively (**Supplementary Figure S1**). Importantly, the peptide encoded by LINC00909 was predicted in a previous study, but lacked experimental support (Ota et al., 2004).

### Significant Dysregulation of 54 Translated Long Non-Coding Ribonucleic Acids Identified in Cancer

Recently, the lncRNA HOXB-AS3-encoded micropeptide has been proven to play an essential role in the regulation of tumorigenesis (Huang et al., 2017); however, little is known of the pathological functions of other lncRNA-encoded micropeptides in cancer. To systematically identify cancer-related lncRNA-encoded micropeptides, we first investigated their composition and abundance in cancer by analyzing 11 genome-wide lncRNA microarray datasets derived from seven cancer types (**Supplementary Table S3**). The lncRNA microarray platform could detect the expression profiles of 110 putative translated lncRNAs. By comparing their expression levels in tumor tissues and corresponding normal tissues, 50 significantly differentially expressed lncRNAs were identified (**Supplementary Table S7**). Of these, six lncRNAs were significantly dysregulated in more than one cancer type (**Supplementary Table S7**).

**Figure 4:**
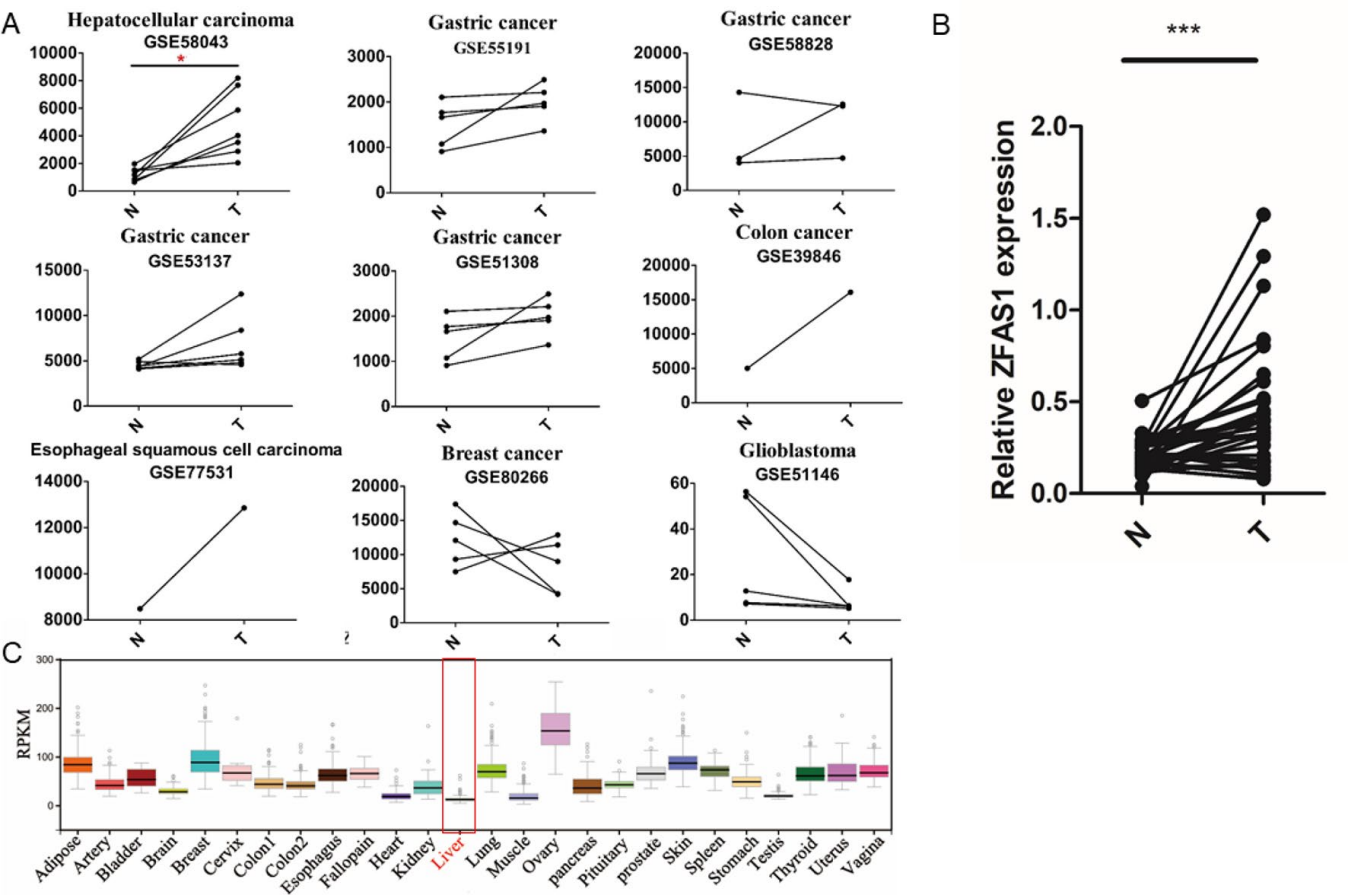
Expression profiles of ZFAS1 in multiple tumor and normal tissues. **(A)** ZFAS1 expression levels in 11 cancer-related long non-coding RNA microarray datasets derived from seven cancer types. Y-axis means the signals detected by the microarray. The red asterisk means that the false positive rate (FDR) is less than 0.1. **(B)** Relative expression levels of ZFAS1 in 32 pairs of hepatocellular carcinoma tumor tissue and corresponding adjacent normal tissue detected by reverse transcription quantitative polymerase chain reaction. ZFAS1 was significantly expressed in tumor tissues (*p-value* = 1.6e−05, paired Wilcoxon rank sum test, n = 32). **(C)** Expression profiles of ZFAS1 in 25 normal tissues downloaded from Genotype-Tissue xpression. ZFAS1 was nearly unexpressed in normal liver tissue. N and T represent adjacent normal tissues and tumor tissues. ***P-value < 0.001.

Interestingly, two of the five experimentally validated translated lncRNAs, ZFAS1 displayed significantly increased expression in HCC tissue (FC = 4.04, FDR = 5.5e−02, paired Wilcoxon rank sum test, **Figure 4A** and **Supplementary Table S7**) and LINC00909 showed significantly decreased expression in gastric cancer tissues (FC = 0.41, FDR = 3.6e−02, paired Wilcoxon rank sum test, **Supplementary Table S7**), respectively. As our lab focused on HCC pathological investigation and previous studies have shown ZFAS1 can exert their functions by lncRNAs in HCC (Dong et al., 2018; Luo et al., 2018), the expression level of ZFAS1 was further validated in 32 pairs of HCC/non-tumor tissue specimens using RT-qPCR. The results showed that ZFAS1 expression was significantly elevated in cancer tissues in agreement with the microarray data (P = 1.6e−05, paired Wilcoxon rank sum test, **Figure 4B**). More importantly, ZFAS1 was nearly undetected in normal liver tissues (**Figure 4C**), indicating that ZFAS1 may have an essential role in HCC tumorigenesis and could serve as a diagnostic marker for HCC.

### ZFAS1 Promotes Cancer Cell Migration

**Figure 5:**
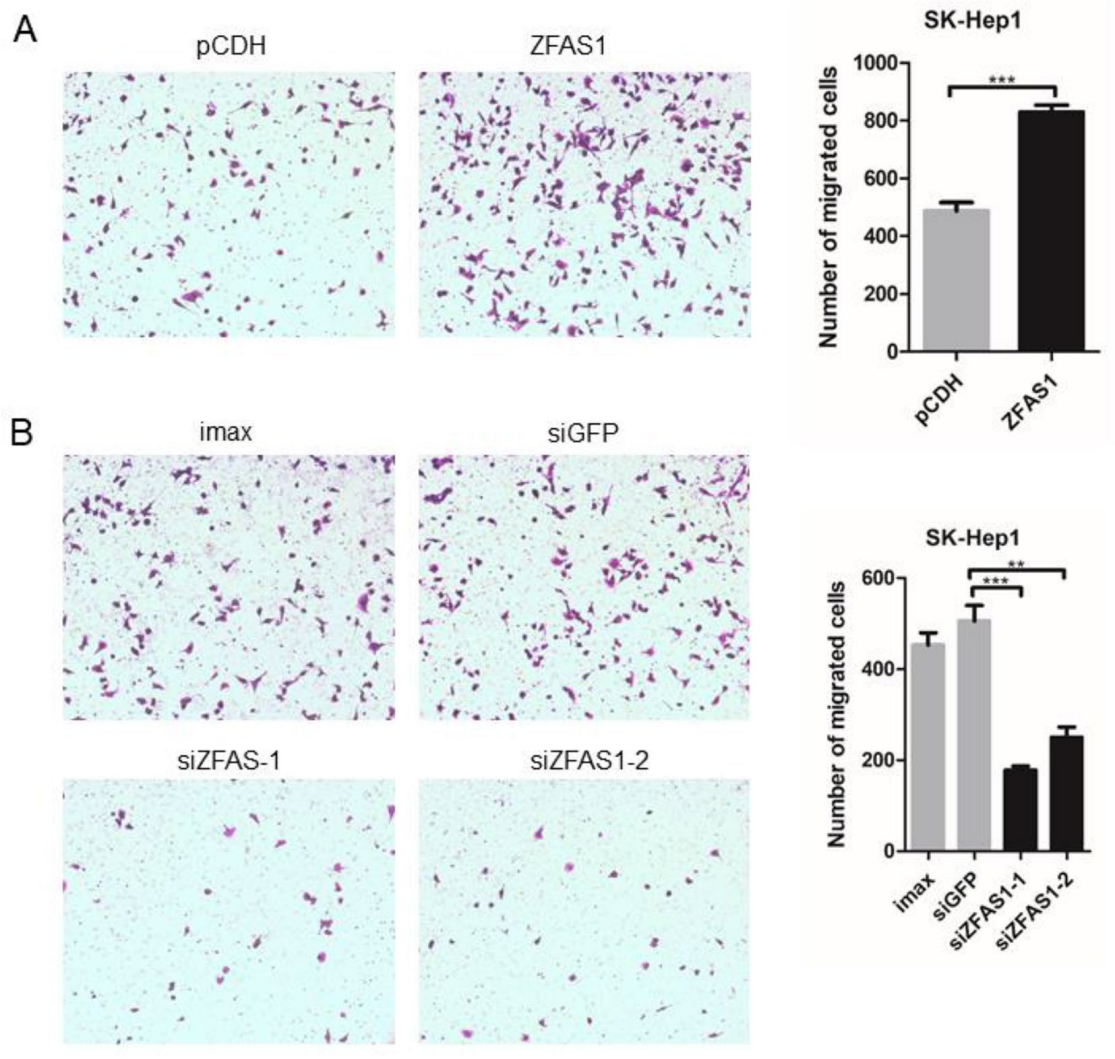
Cell motility changed significantly following ZFAS1 overexpression and knockdown. **(A)** SK-Hep1 cells transfected with ZFAS1 expression plasmid. The cell motility of SK-Hep1 cells was significantly increased following transfection with the ZFAS1 expression plasmid (*p-value* = 9e−04, paired Student’s t-test, n = 3). **(B)** SK-Hep1 cells transfected with small interfering RNAs (siRNAs) of ZFAS1. The cell motility of SK-Hep1 cells decreased significantly following transfection with the two independent siRNAs (siGFP *vs.* siZFAS1-1: *P-value* = 7e−04, siGFP *vs.* siZFAS1-2: *P-value* = 3.2e−03, paired Student’s t-test, n = 3). Imax means cells exposed to Lipofectamine RNAiMAX but not RNA duplexes. siGFP indicates cells transfected with siRNA of green fluorescent protein. **P-value < 0.01, ***P-value < 0.001.

To elucidate the involvement of ZFAS1 in tumorigenesis, the human SK-Hep1 cell line was transfected with pCDH-ZFAS1-ORF or one of two different siRNAs, respectively. The relative RNA expression of ZFAS1 markedly increased and decreased following transfection with the ZFAS1 overexpression plasmid and siRNAs, respectively (overexpression: *P-value* = 6e−04, paired Student’s t-test; siRNA-1: *P-value* = 8.1e−03, siRNA-2: *P-value* = 7.8e−03, Welch’s t-test; **Supplementary Figure S5**). The migratory ability of SK-Hep1 cells transfected with pCDH-ZFAS1-ORF was accelerated compared with the control group (P = 9e−04, paired Student’s t-test, **Figure 5A**), while the migratory ability of SK-Hep1 cells treated with the two different siRNAs was consistently suppressed compared with the controls (siRNA of GFP (siGFP) *vs.* siZFAS1-1: *P-value* = 7e−04, siGFP *vs.* siZFAS1-2: *P-value* = 3.2e−03, paired Student’s t-test, **Figure 5B**). Therefore, the ZFAS1 gene may be a positive regulator of human hepatoma cell migration, with tumor promotion effects.

### Increased Reactive Oxygen Species Production May Correlate With Cell Migration

To clarify the molecular phenotype associated with ZFAS1, we conducted RNA-Seq following ZFAS1 overexpression and knockdown. The expression of 101 and seven genes were significantly downregulated and upregulated in ZFAS1 overexpression cells compared with the controls, respectively. By further analyzing their expression profiles in ZFAS1 knockdown samples, we found that 87 downregulated and 4 upregulated genes showed inverse changes (**Supplementary Table S8**). Next, functional enrichment analysis was performed to investigate the enriched functions of these 91 consistent genes. The results showed that oxidative phosphorylation and ribosome-related pathways were the most enriched GO terms (**Supplementary Figure S6**). From the literature, we found a close relationship between cell migration and the oxidative phosphorylation pathway. Previous studies have revealed that downregulated nicotinamide adenine dinucleotide (NADH) dehydrogenase promotes cell migration by increasing intracellular ROS production (Li et al., 2015a). Furthermore, ROS act as signaling molecules to regulate cell migration (Park et al., 2012; Sena and Chandel, 2012; Liu et al., 2014). Therefore, we speculated that upregulated ZFAS1 increased ROS production by inhibiting NADH dehydrogenase expression to promote cell migration (**Supplementary Figure S7**).

**Figure 6:**
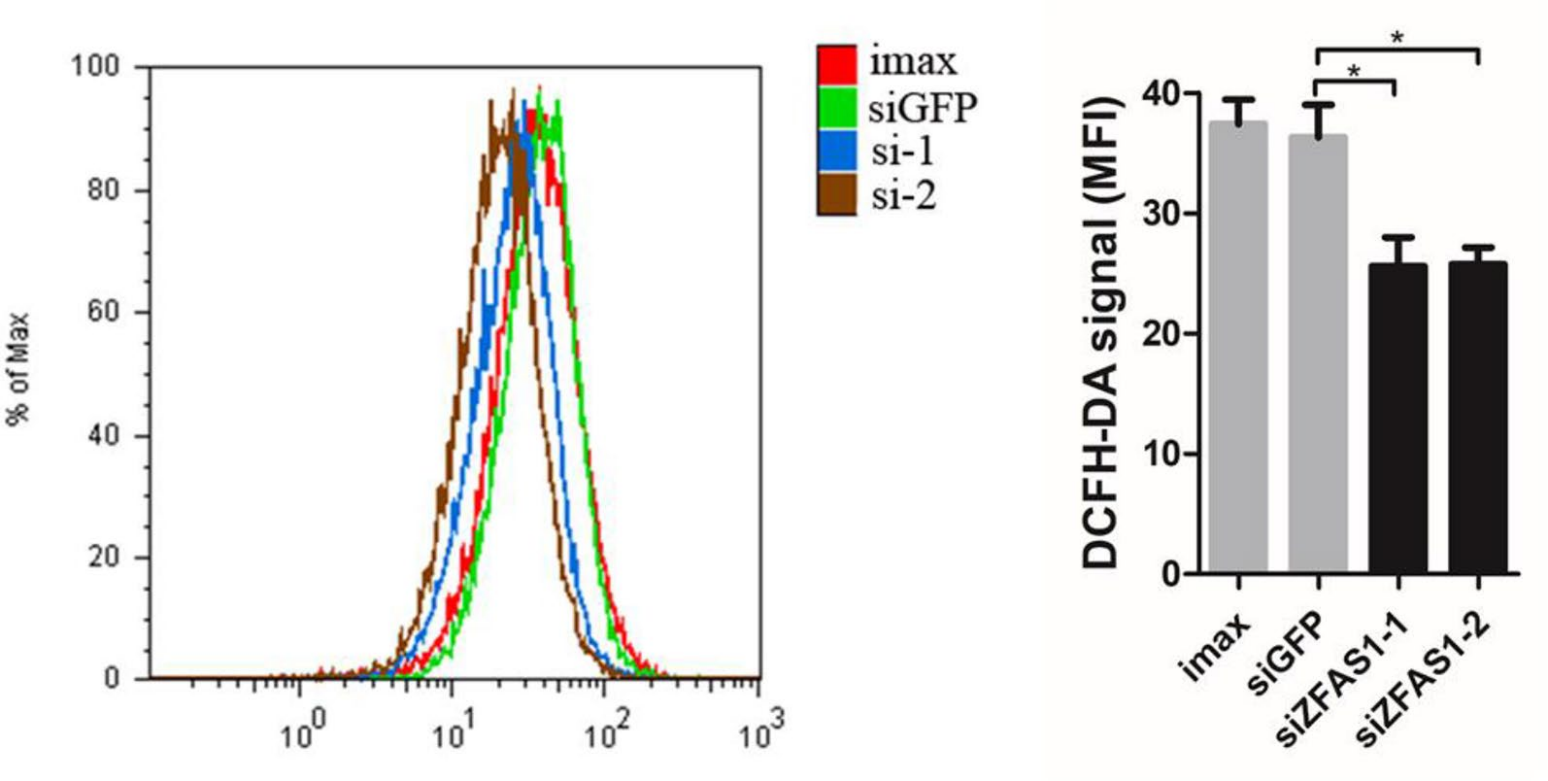
Change in reactive oxygen species (ROS) production following ZFAS1 knockdown. Cellular ROS production was significantly downregulated following transfection of SK-Hep1 cells with two independent siRNAs (siGFP *vs.* siZFAS1-1: *P-value* = 0.022, siGFP *vs.* siZFAS1-2: *P-value* = 0.025, paired Student’s t-test, n = 3). Imax means cells exposed to Lipofectamine RNAiMAX but not RNA duplexes. siGFP indicates cells transfected with small interfering RNAs of green fluorescent protein. *P-value < 0.05.

According to the literature results, we first detected ROS production in ZFAS1 knockdown cells using flow cytometry. ROS levels were remarkably down-regulated in ZFAS1 knockdown samples, indicating that ZFAS1 positively correlated with ROS production (**Figure 6**). As previous studies have proven that the down-regulated NADH dehydrogenase promotes cell migration by increasing intracellular ROS production, we further explored the correlation between NADH dehydrogenase and ROS production. Our results showed that the relative RNA expression of NADH dehydrogenase (NDUFA6, NDUFA7, NDUFB4, and NDUFB11) was markedly decreased following transfection with the ZFAS1 expression plasmid and showed reverse changes in the RNA interference samples (**Supplementary Table S5**). Furthermore, we validated whether NADH dehydrogenase expression negatively correlated with ROS production. Because SOD2 is a ROS marker reflecting its levels, we calculated the correlation between the expression of SOD2 and four NADH dehydrogenases using HCC TCGA data. The expression of NDUFA6, NDUFB4, and NDUFB11 showed significant negative correlations with SOD2, indicating a significant negative correlation between ROS production and NADH dehydrogenase (NDUFA6: r = −0.24, p = 3.82e−06; NDUFB4: r = −0.24, p = 4.41e−06; NDUFB11: r = −0.2, p = 1e−04, **Supplementary Figure S8**). Therefore, we propose that upregulated ZFAS1 promotes cancer cell migration *via* elevating cellular ROS production by repressing the expression of NADH dehydrogenases, including NDUFA6, NDUFB4, and NDUFB11 (**Supplementary Figure S7**).

## Discussion

We systematically analyzed the functional roles of lncRNA-encoded micropeptides in cancer by incorporating multiple high throughput datasets, such as RPF-Seq, genome-wide lncRNA microarray, and RNA-Seq. Hundreds of translated smORFs were identified and numerous significantly differentially expressed genes were observed in multiple cancer types, supporting the essential roles of translated smORFs in tumorigenesis.

Compared to other studies of lncRNA-encoded translated smORFs, our study offers several advantages. First, more translated smORFs were predicted by our classifiers and 537 translated smORFs were identified in our study (Bazzini et al., 2014). To identify translated smORFs as much as possible, three ribosome features derived from two RPF-Seq datasets combined with four machine learning methods were used to construct the classifiers. The number of our predicted translated smORFs was more than previous studies. Second, apart from predicting translated smORFs based on classification models, two experimental methods (construct generation and MS) were implemented to validate the protein-coding potential of lncRNAs (Calviello et al., 2016). Experimental validation is critical to assess the performance of classification models. Third, we not only identified translated smORFs, but also further explored their composition and abundance in seven cancer types to find functional translated lncRNAs. By analyzing 11 genome-wide lncRNA microarrays, we found that substantial numbers of putative translated lncRNAs were significantly differentially expressed in cancer, indicating their important roles in tumorigenesis. Fourth, one cancer-related translated lncRNA, ZFAS1, was found to play important roles in promoting cancer cell migration and affecting cell metabolism in HCC (**Figures 4** and **5**), which indicated that our method could identify cancer-related translated lncRNAs. Therefore, all putative translated lncRNAs and their cancer-related information were presented in the supplementary material to facilitate the investigation of translated smORFs in cancer pathogenesis.

Despite these advantages, this study had several limitations. A previous study revealed that some translated smORFs with non-traditional start codons (CUG, GUG) have essential functions (Ingolia et al., 2011). In this study, we only defined ORFs with the traditional start codon AUG, possibly excluding translated smORFs with non-traditional start codons. Moreover, previous studies have also shown that some translated ORFs existed in 5′ and 3′ UTRs, which were defined as upstream and downstream coding ORFs (Oyama et al., 2004; Fritsch et al., 2012; Chew et al., 2013). In our study, we first identified all ORFs in 5′ and 3′ UTRs, and then filtered the potential upstream and downstream coding ORFs according to literature (Vilela and Mccarthy, 2003; Oyama et al., 2004; Fritsch et al., 2012; Chew et al., 2013; Guttman et al., 2013; Slavoff et al., 2013; Calviello et al., 2016). The remaining ORFs were used as negative controls. Therefore, our method could not identify the translated ORFs derived from 5′ and 3′ UTRs. In addition, more evidence was needed to prove the roles of ZFAS1 in metastasis through protein form.

ZFAS1 may participate in multiple biological processes to regulate tumorigenesis and metastasis. Here, we found that one validated coding smORF derived from ZFAS1 was upregulated in HCC tumor tissue and nearly unexpressed in normal liver tissue. More importantly, ZFAS1 promoted cancer cell migration and ROS production in HCCs to participate in tumorigenesis (**Figures 4** and **5**). By analyzing the ZFAS1 gene, translated smORFS were only concealed in the transcript of ZFAS1 (ENST00000458653). Based on the literature, we found other transcripts of ZFAS1 with important roles in regulating tumorigenesis (Li et al., 2015b; Wang and Xing, 2016; Zhou et al., 2016; Lv et al., 2017). For example, in glioma and gastric cancers, the upregulation of ZFAS1 could enhance the epithelial mesenchymal transition (EMT) (Zhou et al., 2016; Lv et al., 2017). Meanwhile, in colorectal cancer and HCC, ZFAS1 regulated tumor metastasis (Li et al., 2015b; Wang and Xing, 2016). Therefore, ZFAS1 may be involved in other aspects of cancer hallmarks apart from cell mobility.

In summary, we identified 537 putative translated smORFs derived from lncRNAs using newly developed classifiers. Moreover, we identified 50 cancer-related translated lncRNAs by exploring their composition and abundance in cancer. Finally, we found that the experimentally validated translated lncRNA ZFSA1 promoted cancer cell migration by elevating cellular ROS production *via* the expression downregulation of NADH dehydrogenase expression (NDUFA6, NDUFB4, and NDUFB11). These findings help further clarify our understanding of the critical roles of smORFs in physiological and pathological processes, especially in cancer.

## Data Availability Statement

The RNA-sequencing data are available on GEO (GSE104226).

## Author Contributions

X-XY and Y-SW designed and supervised the study. Z-WG analyzed and interpreted the data and prepared the manuscript. YM and CX designed the study, provided samples, and interpreted clinical data. NZ, ML, X-MZ, C-LZ, KL, and T-CL analyzed and interpreted the data. All authors vouched for the respective data and analysis, reviewed and approved the final version, and agreed to publish the manuscript.

## Funding

This study was funded by National Science Foundation for Young Scientists of China (81802435), China Postdoctoral Science Foundation funded project (2016M602486, 2019T120742), Science and Technology Program of Guangdong (2015B020233009, 2015A030401040).

## Conflict of Interest

The authors declare that the research was conducted in the absence of any commercial or financial relationships that could be construed as a potential conflict of interest.
