# Supplementary material for: Translated Long Non-Coding Ribonucleic Acid ZFAS1 Promotes Cancer Cell Migration by Elevating Reactive Oxygen Species Production in Hepatocellular Carcinoma
Source: Front Genet. 2019 Nov 12;10:1111. doi: 10.3389/fgene.2019.01111 (PMC6861293; doi:10.3389/fgene.2019.01111)
Supplement: Supplementary file 1 [file Table_1.docx]

**Supplementary Material**

Translated lncRNA ZFAS1 promotes cancer cell migration by elevating reactive oxygen species production in hepatocellular carcinoma

Zhi-Wei Guo^1,*^, Yu Meng^2,*^, Xiang-Ming Zhai^1,*^, Chen Xie^2^, Na Zhao^4^, Min Li^1^, Chun-Lian Zhou^1^, Kun Li^1^, Xue-Xi Yang^1, †^, Ying-Song Wu^1, †^

^1^ Institute of Antibody Engineering, School of Laboratory Medicine and Biotechnology, Southern Medical University, Guangzhou, P.R China

^2^ Key Laboratory of Gene Engineering of the Ministry of Education, School of Life Sciences, Sun Yat-sen University, Guangzhou, P. R. China

^3^ Key Laboratory of Liver Disease of Guangdong Province, The Third Affiliated Hospital of Sun Yat-sen University, Guangzhou, P. R. China

* co-first authors: Zhi-Wei Guo and Yu Meng

**Supplementary Table**

**Supplementary Table S1.** The number of ORFs used in classifier construction

| Cell line | Total positive | Total negative | Training positive | Training negative | Validation positive | Validation negative | Prediction |
| --- | --- | --- | --- | --- | --- | --- | --- |
| U2OS | 4149 | 6889 | 2904 | 4822 | 1245 | 2067 | 5114 |
| HeLa | 3968 | 5555 | 2778 | 3889 | 1190 | 1666 | 3654 |

Total positive and total negative means the number of all coding ORFs and non-coding ORFs used in classifier construction, respectively. Training positive and total negative means the number of coding ORFs and non-coding ORFs used in training classifiers, respectively. Validation positive and total negative means the number of coding ORFs and non-coding ORFs used in validating the performance of classifiers, respectively. Prediction means the number of smORFs assessed by the classifiers.

**Supplementary Table S2.** Primers used for cloning putative translated smORFs derived from lncRNAs

| ID | Sense Primer (5' - 3') | Antisense Primer (5' - 3') |
| --- | --- | --- |
| ZFAS1  (ENST00000458653) | CGAGAATTCCGGAGCCGGCGGGCTC | TCCTCGCCCTTGCTCACGGAGATCGAAGGTTGTAG |
| RP11-879F14.2  (ENST00000586949) | CGAGAATTCCGCCAAGAGCGCACAGAC | TCCTCGCCCTTGCTCACGTTCTCCTTCATTTCC |
| SNHG8  (ENST00000602483) | CGGGATCCGGCCTTTCACATTCGGG | CGCCCTTGCTCACTTCGGAACACCCGTTTCCCC |
| RP4-614O4.11  (ENST00000444717) | CGAGAATTCGGAAGCTACTGTGTC | TCCTCGCCCTTGCTCACAACACTTATCCTCAGTG |
| RP11-554I8.2  (ENST00000417112) | CGAGAATTCCCCTCCGTCTCCTTGACTC | TCCTCGCCCTTGCTCACTTTAAAAGGTGTACTGGACCGG |

Primers were used to clone the 5′UTR-ORF of five selected translated smORFs derived from lncRNAs.

**Supplementary Table S3.** LncRNA microarrays used for evaluating lncRNA abundance and composition

| Cancer type | GEO ID | PMID | Number |
| --- | --- | --- | --- |
| Hepatocellular carcinoma | GSE55191 | 26054679 | 6 |
| Hepatocellular carcinoma | GSE58043 | 27530352 | 14 |
| Gastric cancer | GSE58828 | 26549025 | 6 |
| Gastric cancer | GSE54835 | 26863570 | 4 |
| Gastric cancer | GSE51308 | 25973314 | 10 |
| Gastric cancer | GSE53137 | 25769450 | 12 |
| Bladder cancer | GSE83955 | 27465044 | 2 |
| Breast cancer | GSE80266 | 27228351 | 10 |
| Esophageal squamous cell carcinoma | GSE77531 | 27058444 | 2 |
| Glioblastoma | GSE51146 | 28337377 | 10 |
| Colorectal cancer | GSE39846 | 25663692 | 2 |

Number = sample number used in each microarray dataset.

**Supplementary Table S4.** Putative translated smORFs predicted by our classifiers.

| **ID** | **start** | **end** | **length** | **ensembl** | **gene** | **type** |
| --- | --- | --- | --- | --- | --- | --- |
| smORF_1 | 89 | 346 | 258 | ENST00000502171 | CTD-2366F13.1 | antisense |
| smORF_2 | 32 | 289 | 258 | ENST00000499459 | CTD-2366F13.1 | antisense |
| smORF_3 | 54 | 284 | 231 | ENST00000512301 | CTD-2366F13.1 | antisense |
| smORF_4 | 4 | 189 | 186 | ENST00000445681 | GS1-124K5.4 | lincRNA |
| smORF_5 | 202 | 396 | 195 | ENST00000447221 | SNHG7 | antisense |
| smORF_6 | 7 | 171 | 165 | ENST00000449713 | AP001065.15 | lincRNA |
| smORF_7 | 20 | 247 | 228 | ENST00000576606 | DLGAP1-AS1 | antisense |
| smORF_8 | 483 | 638 | 156 | ENST00000523225 | RP11-115C21.2 | antisense |
| smORF_9 | 412 | 567 | 156 | ENST00000606853 | RP11-115C21.2 | antisense |
| smORF_10 | 418 | 573 | 156 | ENST00000500118 | RP11-115C21.2 | antisense |
| smORF_11 | 206 | 343 | 138 | ENST00000445520 | AC007246.3 | antisense |
| smORF_12 | 348 | 521 | 174 | ENST00000445427 | PRKCQ-AS1 | lincRNA |
| smORF_13 | 410 | 643 | 234 | ENST00000585075 | RP11-649A18.12 | antisense |
| smORF_14 | 397 | 705 | 309 | ENST00000540684 | LINC00944 | lincRNA |
| smORF_15 | 2884 | 3006 | 123 | ENST00000534336 | MALAT1 | lincRNA |
| smORF_16 | 1601 | 1723 | 123 | ENST00000544868 | MALAT1 | lincRNA |
| smORF_17 | 274 | 426 | 153 | ENST00000434063 | HOTAIRM1 | antisense |
| smORF_18 | 209 | 496 | 288 | ENST00000561547 | RP11-22P6.3 | antisense |
| smORF_19 | 20 | 100 | 81 | ENST00000434872 | RP11-452F19.3 | lincRNA |
| smORF_20 | 286 | 477 | 192 | ENST00000498624 | RP11-221J22.2 | lincRNA |
| smORF_21 | 286 | 450 | 165 | ENST00000533253 | CTD-2523D13.2 | antisense |
| smORF_22 | 185 | 343 | 159 | ENST00000598595 | RP11-381O7.3 | lincRNA |
| smORF_23 | 143 | 382 | 240 | ENST00000455011 | RP3-460G2.2 | lincRNA |
| smORF_24 | 180 | 365 | 186 | ENST00000434627 | RP11-195F19.9 | antisense |
| smORF_25 | 181 | 276 | 96 | ENST00000533767 | NAV2-AS2 | antisense |
| smORF_26 | 110 | 274 | 165 | ENST00000566675 | LOXL1-AS1 | antisense |
| smORF_27 | 73 | 258 | 186 | ENST00000465215 | RP11-221J22.1 | lincRNA |
| smORF_28 | 619 | 810 | 192 | ENST00000602845 | NCBP2-AS2 | lincRNA |
| smORF_29 | 1616 | 1786 | 171 | ENST00000554360 | RP11-349A22.5 | antisense |
| smORF_30 | 109 | 213 | 105 | ENST00000424345 | RP11-262H14.1 | lincRNA |
| smORF_31 | 113 | 217 | 105 | ENST00000427509 | RP11-262H14.1 | lincRNA |
| smORF_32 | 176 | 280 | 105 | ENST00000452184 | RP11-262H14.1 | lincRNA |
| smORF_33 | 154 | 285 | 132 | ENST00000442007 | RP11-398K22.12 | antisense |
| smORF_34 | 203 | 322 | 120 | ENST00000414030 | LINC00152 | lincRNA |
| smORF_35 | 119 | 325 | 207 | ENST00000609228 | MAPKAPK5-AS1 | lincRNA |
| smORF_36 | 1315 | 1521 | 207 | ENST00000456429 | MAPKAPK5-AS1 | lincRNA |
| smORF_37 | 123 | 329 | 207 | ENST00000609983 | MAPKAPK5-AS1 | lincRNA |
| smORF_38 | 1315 | 1521 | 207 | ENST00000428207 | MAPKAPK5-AS1 | lincRNA |
| smORF_39 | 3541 | 3708 | 168 | ENST00000534336 | MALAT1 | lincRNA |
| smORF_40 | 2258 | 2425 | 168 | ENST00000544868 | MALAT1 | lincRNA |
| smORF_41 | 117 | 404 | 288 | ENST00000483840 | RP11-221J22.2 | lincRNA |
| smORF_42 | 280 | 567 | 288 | ENST00000465215 | RP11-221J22.1 | lincRNA |
| smORF_43 | 191 | 301 | 111 | ENST00000412427 | RP11-380J14.1 | lincRNA |
| smORF_44 | 45 | 269 | 225 | ENST00000483578 | RP11-2C24.4 | lincRNA |
| smORF_45 | 269 | 547 | 279 | ENST00000591384 | CTD-2357A8.3 | lincRNA |
| smORF_46 | 154 | 432 | 279 | ENST00000586185 | CTD-2357A8.3 | lincRNA |
| smORF_47 | 138 | 410 | 273 | ENST00000359941 | TP53TG1 | lincRNA |
| smORF_48 | 86 | 358 | 273 | ENST00000610086 | TP53TG1 | lincRNA |
| smORF_49 | 698 | 841 | 144 | ENST00000451775 | AC099850.1 | antisense |
| smORF_50 | 243 | 500 | 258 | ENST00000417559 | RP11-137H2.4 | antisense |
| smORF_51 | 41 | 283 | 243 | ENST00000357401 | AP001597.1 | antisense |
| smORF_52 | 500 | 622 | 123 | ENST00000490375 | RP11-274H2.3 | antisense |
| smORF_53 | 61 | 207 | 147 | ENST00000510137 | CTD-2263F21.1 | antisense |
| smORF_54 | 51 | 125 | 75 | ENST00000412092 | RP11-417J8.3 | lincRNA |
| smORF_55 | 75 | 149 | 75 | ENST00000400755 | RP11-417J8.3 | lincRNA |
| smORF_56 | 1206 | 1427 | 222 | ENST00000566733 | RP1-140K8.5 | lincRNA |
| smORF_57 | 80 | 286 | 207 | ENST00000499499 | DNAJC3-AS1 | lincRNA |
| smORF_58 | 75 | 155 | 81 | ENST00000606011 | DNAJC3-AS1 | lincRNA |
| smORF_59 | 1154 | 1240 | 87 | ENST00000421692 | ZNRD1-AS1 | antisense |
| smORF_60 | 978 | 1061 | 84 | ENST00000437417 | ZNRD1-AS1 | antisense |
| smORF_61 | 261 | 344 | 84 | ENST00000376797 | ZNRD1-AS1 | antisense |
| smORF_62 | 458 | 541 | 84 | ENST00000422224 | ZNRD1-AS1 | antisense |
| smORF_63 | 439 | 522 | 84 | ENST00000420251 | ZNRD1-AS1 | antisense |
| smORF_64 | 29 | 145 | 117 | ENST00000440377 | RP11-337C18.8 | antisense |
| smORF_65 | 245 | 298 | 54 | ENST00000522817 | GS1-251I9.4 | antisense |
| smORF_66 | 311 | 364 | 54 | ENST00000524003 | GS1-251I9.4 | antisense |
| smORF_67 | 255 | 476 | 222 | ENST00000590513 | RP11-400F19.6 | antisense |
| smORF_68 | 188 | 451 | 264 | ENST00000581996 | RP11-17M16.2 | antisense |
| smORF_69 | 122 | 178 | 57 | ENST00000488425 | RP11-88I21.2 | lincRNA |
| smORF_70 | 583 | 636 | 54 | ENST00000558174 | RP11-752G15.6 | antisense |
| smORF_71 | 532 | 585 | 54 | ENST00000559366 | RP11-752G15.6 | antisense |
| smORF_72 | 650 | 703 | 54 | ENST00000561107 | RP11-752G15.6 | antisense |
| smORF_73 | 253 | 420 | 168 | ENST00000538731 | RP13-977J11.2 | lincRNA |
| smORF_74 | 173 | 283 | 111 | ENST00000441217 | AC073046.25 | lincRNA |
| smORF_75 | 102 | 233 | 132 | ENST00000473550 | LINC00883 | lincRNA |
| smORF_76 | 131 | 262 | 132 | ENST00000466734 | LINC00883 | lincRNA |
| smORF_77 | 131 | 262 | 132 | ENST00000463143 | LINC00883 | lincRNA |
| smORF_78 | 131 | 262 | 132 | ENST00000490441 | LINC00883 | lincRNA |
| smORF_79 | 373 | 576 | 204 | ENST00000569214 | RP11-872J21.3 | antisense |
| smORF_80 | 94 | 297 | 204 | ENST00000539116 | RP11-221N13.3 | lincRNA |
| smORF_81 | 50 | 145 | 96 | ENST00000515218 | RP11-129M6.1 | lincRNA |
| smORF_82 | 469 | 726 | 258 | ENST00000608279 | RP11-725P16.2 | lincRNA |
| smORF_83 | 126 | 242 | 117 | ENST00000429600 | TAPSAR1 | lincRNA |
| smORF_84 | 297 | 413 | 117 | ENST00000564963 | LOXL1-AS1 | antisense |
| smORF_85 | 202 | 318 | 117 | ENST00000565756 | LOXL1-AS1 | antisense |
| smORF_86 | 342 | 611 | 270 | ENST00000556354 | RP11-66N24.4 | antisense |
| smORF_87 | 115 | 384 | 270 | ENST00000553985 | RP11-66N24.4 | antisense |
| smORF_88 | 140 | 319 | 180 | ENST00000398043 | RSBN1L-AS1 | lincRNA |
| smORF_89 | 140 | 319 | 180 | ENST00000440088 | RSBN1L-AS1 | lincRNA |
| smORF_90 | 128 | 307 | 180 | ENST00000447009 | RSBN1L-AS1 | lincRNA |
| smORF_91 | 306 | 464 | 159 | ENST00000418837 | RP11-557H15.4 | lincRNA |
| smORF_92 | 289 | 345 | 57 | ENST00000436383 | LINC00707 | lincRNA |
| smORF_93 | 491 | 658 | 168 | ENST00000424349 | FGD5-AS1 | antisense |
| smORF_94 | 466 | 633 | 168 | ENST00000440079 | FGD5-AS1 | antisense |
| smORF_95 | 482 | 649 | 168 | ENST00000426200 | FGD5-AS1 | antisense |
| smORF_96 | 225 | 392 | 168 | ENST00000417835 | FGD5-AS1 | antisense |
| smORF_97 | 8 | 109 | 102 | ENST00000577743 | RP11-401O9.3 | antisense |
| smORF_98 | 78 | 215 | 138 | ENST00000550680 | RP11-1103G16.1 | antisense |
| smORF_99 | 5 | 196 | 192 | ENST00000565764 | CTA-445C9.14 | antisense |
| smORF_100 | 23 | 214 | 192 | ENST00000566814 | CTA-445C9.14 | antisense |
| smORF_101 | 362 | 625 | 264 | ENST00000571449 | RP11-473M20.14 | antisense |
| smORF_102 | 250 | 393 | 144 | ENST00000576590 | RP11-473M20.14 | antisense |
| smORF_103 | 206 | 442 | 237 | ENST00000534671 | SBF2-AS1 | antisense |
| smORF_104 | 264 | 419 | 156 | ENST00000498905 | SBF2-AS1 | antisense |
| smORF_105 | 81 | 278 | 198 | ENST00000565633 | SNAI3-AS1 | antisense |
| smORF_106 | 273 | 311 | 39 | ENST00000607531 | RP11-557H15.4 | lincRNA |
| smORF_107 | 430 | 648 | 219 | ENST00000440377 | RP11-337C18.8 | antisense |
| smORF_108 | 10 | 141 | 132 | ENST00000562739 | LOXL1-AS1 | antisense |
| smORF_109 | 155 | 283 | 129 | ENST00000564194 | LOXL1-AS1 | antisense |
| smORF_110 | 14 | 280 | 267 | ENST00000560395 | NR2F2-AS1 | antisense |
| smORF_111 | 56 | 289 | 234 | ENST00000457390 | RP11-14N7.2 | lincRNA |
| smORF_112 | 720 | 920 | 201 | ENST00000429940 | LINC00839 | lincRNA |
| smORF_113 | 719 | 919 | 201 | ENST00000424751 | LINC00839 | lincRNA |
| smORF_114 | 843 | 1031 | 189 | ENST00000508564 | RP11-834C11.4 | lincRNA |
| smORF_115 | 56 | 181 | 126 | ENST00000608792 | LINC00702 | lincRNA |
| smORF_116 | 72 | 173 | 102 | ENST00000598518 | AC006262.5 | lincRNA |
| smORF_117 | 93 | 194 | 102 | ENST00000599817 | AC006262.5 | lincRNA |
| smORF_118 | 44 | 145 | 102 | ENST00000594027 | AC006262.5 | lincRNA |
| smORF_119 | 93 | 194 | 102 | ENST00000595673 | AC006262.5 | lincRNA |
| smORF_120 | 177 | 302 | 126 | ENST00000422199 | LINC00162 | lincRNA |
| smORF_121 | 41 | 112 | 72 | ENST00000606039 | RP11-557H15.4 | lincRNA |
| smORF_122 | 29 | 112 | 84 | ENST00000510469 | CTD-2263F21.1 | antisense |
| smORF_123 | 74 | 169 | 96 | ENST00000529369 | RP11-660L16.2 | antisense |
| smORF_124 | 315 | 479 | 165 | ENST00000502162 | LINC00847 | lincRNA |
| smORF_125 | 561 | 824 | 264 | ENST00000295549 | LINC01116 | lincRNA |
| smORF_126 | 373 | 465 | 93 | ENST00000380722 | RP11-497E19.1 | lincRNA |
| smORF_127 | 185 | 265 | 81 | ENST00000568087 | LOXL1-AS1 | antisense |
| smORF_128 | 314 | 394 | 81 | ENST00000567644 | LOXL1-AS1 | antisense |
| smORF_129 | 183 | 263 | 81 | ENST00000565416 | LOXL1-AS1 | antisense |
| smORF_130 | 203 | 283 | 81 | ENST00000562965 | LOXL1-AS1 | antisense |
| smORF_131 | 111 | 191 | 81 | ENST00000568229 | LOXL1-AS1 | antisense |
| smORF_132 | 388 | 468 | 81 | ENST00000567257 | LOXL1-AS1 | antisense |
| smORF_133 | 122 | 202 | 81 | ENST00000562130 | LOXL1-AS1 | antisense |
| smORF_134 | 417 | 533 | 117 | ENST00000565523 | RP11-244B22.11 | lincRNA |
| smORF_135 | 264 | 317 | 54 | ENST00000448513 | RP11-395B7.4 | antisense |
| smORF_136 | 294 | 347 | 54 | ENST00000441882 | RP11-395B7.4 | antisense |
| smORF_137 | 493 | 783 | 291 | ENST00000608317 | PAXIP1-AS1 | lincRNA |
| smORF_138 | 543 | 626 | 84 | ENST00000567257 | LOXL1-AS1 | antisense |
| smORF_139 | 216 | 299 | 84 | ENST00000562739 | LOXL1-AS1 | antisense |
| smORF_140 | 277 | 360 | 84 | ENST00000562130 | LOXL1-AS1 | antisense |
| smORF_141 | 358 | 441 | 84 | ENST00000564194 | LOXL1-AS1 | antisense |
| smORF_142 | 338 | 421 | 84 | ENST00000565416 | LOXL1-AS1 | antisense |
| smORF_143 | 246 | 347 | 102 | ENST00000509921 | LINC00847 | lincRNA |
| smORF_144 | 426 | 527 | 102 | ENST00000501937 | LINC00847 | lincRNA |
| smORF_145 | 469 | 510 | 42 | ENST00000567644 | LOXL1-AS1 | antisense |
| smORF_146 | 6 | 47 | 42 | ENST00000565689 | LOXL1-AS1 | antisense |
| smORF_147 | 821 | 1030 | 210 | ENST00000552576 | RP11-644F5.11 | antisense |
| smORF_148 | 491 | 733 | 243 | ENST00000590677 | LINC00662 | lincRNA |
| smORF_149 | 7 | 228 | 222 | ENST00000561521 | LINC00662 | lincRNA |
| smORF_150 | 325 | 360 | 36 | ENST00000447639 | AC007040.7 | antisense |
| smORF_151 | 921 | 1160 | 240 | ENST00000603175 | LBX2-AS1 | antisense |
| smORF_152 | 2751 | 2855 | 105 | ENST00000468186 | SETD5-AS1 | antisense |
| smORF_153 | 148 | 252 | 105 | ENST00000520629 | SETD5-AS1 | antisense |
| smORF_154 | 480 | 584 | 105 | ENST00000522221 | SETD5-AS1 | antisense |
| smORF_155 | 206 | 310 | 105 | ENST00000523354 | SETD5-AS1 | antisense |
| smORF_156 | 130 | 234 | 105 | ENST00000520396 | SETD5-AS1 | antisense |
| smORF_157 | 489 | 593 | 105 | ENST00000521609 | SETD5-AS1 | antisense |
| smORF_158 | 136 | 228 | 93 | ENST00000498199 | SETD5-AS1 | antisense |
| smORF_159 | 76 | 168 | 93 | ENST00000521609 | SETD5-AS1 | antisense |
| smORF_160 | 226 | 318 | 93 | ENST00000469846 | SETD5-AS1 | antisense |
| smORF_161 | 140 | 232 | 93 | ENST00000521708 | SETD5-AS1 | antisense |
| smORF_162 | 102 | 194 | 93 | ENST00000480904 | SETD5-AS1 | antisense |
| smORF_163 | 67 | 159 | 93 | ENST00000522221 | SETD5-AS1 | antisense |
| smORF_164 | 47 | 238 | 192 | ENST00000442316 | AC074363.1 | lincRNA |
| smORF_165 | 123 | 206 | 84 | ENST00000425884 | RP11-418J17.1 | antisense |
| smORF_166 | 94 | 177 | 84 | ENST00000440150 | RP11-418J17.1 | antisense |
| smORF_167 | 299 | 460 | 162 | ENST00000335142 | KTN1-AS1 | antisense |
| smORF_168 | 189 | 350 | 162 | ENST00000535211 | KTN1-AS1 | antisense |
| smORF_169 | 185 | 277 | 93 | ENST00000531263 | RP11-535A19.2 | antisense |
| smORF_170 | 318 | 410 | 93 | ENST00000529719 | RP11-535A19.2 | antisense |
| smORF_171 | 526 | 765 | 240 | ENST00000535639 | CD27-AS1 | antisense |
| smORF_172 | 250 | 405 | 156 | ENST00000606697 | NNT-AS1 | antisense |
| smORF_173 | 124 | 267 | 144 | ENST00000503484 | NNT-AS1 | antisense |
| smORF_174 | 70 | 372 | 303 | ENST00000500682 | RP11-277P12.20 | antisense |
| smORF_175 | 202 | 405 | 204 | ENST00000416970 | SNHG7 | antisense |
| smORF_176 | 202 | 405 | 204 | ENST00000414282 | SNHG7 | antisense |
| smORF_177 | 119 | 253 | 135 | ENST00000452399 | RP11-14N7.2 | lincRNA |
| smORF_178 | 1025 | 1315 | 291 | ENST00000548978 | LBX2-AS1 | antisense |
| smORF_179 | 1257 | 1547 | 291 | ENST00000603175 | LBX2-AS1 | antisense |
| smORF_180 | 219 | 374 | 156 | ENST00000554614 | RP4-647C14.2 | antisense |
| smORF_181 | 14 | 172 | 159 | ENST00000411439 | AL022344.4 | lincRNA |
| smORF_182 | 136 | 390 | 255 | ENST00000501177 | CRNDE | lincRNA |
| smORF_183 | 115 | 330 | 216 | ENST00000499732 | NEAT1 | lincRNA |
| smORF_184 | 74 | 289 | 216 | ENST00000601801 | NEAT1 | lincRNA |
| smORF_185 | 91 | 306 | 216 | ENST00000501122 | NEAT1 | lincRNA |
| smORF_186 | 220 | 300 | 81 | ENST00000503882 | CTC-436P18.3 | lincRNA |
| smORF_187 | 67 | 147 | 81 | ENST00000511794 | CTC-436P18.3 | lincRNA |
| smORF_188 | 1536 | 1655 | 120 | ENST00000534336 | MALAT1 | lincRNA |
| smORF_189 | 253 | 372 | 120 | ENST00000544868 | MALAT1 | lincRNA |
| smORF_190 | 160 | 411 | 252 | ENST00000489616 | SETD5-AS1 | antisense |
| smORF_191 | 143 | 394 | 252 | ENST00000521267 | SETD5-AS1 | antisense |
| smORF_192 | 210 | 461 | 252 | ENST00000481221 | SETD5-AS1 | antisense |
| smORF_193 | 175 | 426 | 252 | ENST00000494680 | SETD5-AS1 | antisense |
| smORF_194 | 91 | 342 | 252 | ENST00000467069 | SETD5-AS1 | antisense |
| smORF_195 | 143 | 394 | 252 | ENST00000519043 | SETD5-AS1 | antisense |
| smORF_196 | 155 | 406 | 252 | ENST00000522525 | SETD5-AS1 | antisense |
| smORF_197 | 116 | 298 | 183 | ENST00000424349 | FGD5-AS1 | antisense |
| smORF_198 | 114 | 296 | 183 | ENST00000440079 | FGD5-AS1 | antisense |
| smORF_199 | 170 | 400 | 231 | ENST00000504032 | UGDH-AS1 | antisense |
| smORF_200 | 301 | 438 | 138 | ENST00000559432 | CRNDE | lincRNA |
| smORF_201 | 195 | 335 | 141 | ENST00000558952 | CRNDE | lincRNA |
| smORF_202 | 138 | 344 | 207 | ENST00000597953 | RP11-91G21.1 | lincRNA |
| smORF_203 | 266 | 550 | 285 | ENST00000568229 | LOXL1-AS1 | antisense |
| smORF_204 | 507 | 578 | 72 | ENST00000540066 | TOPORS-AS1 | antisense |
| smORF_205 | 178 | 369 | 192 | ENST00000551421 | RP11-620J15.3 | lincRNA |
| smORF_206 | 395 | 595 | 201 | ENST00000550853 | LINC00094 | antisense |
| smORF_207 | 442 | 642 | 201 | ENST00000430633 | LINC00094 | antisense |
| smORF_208 | 18 | 254 | 237 | ENST00000442982 | PSMD5-AS1 | antisense |
| smORF_209 | 122 | 358 | 237 | ENST00000586907 | PSMD5-AS1 | antisense |
| smORF_210 | 112 | 246 | 135 | ENST00000523341 | RP11-513O17.2 | lincRNA |
| smORF_211 | 172 | 267 | 96 | ENST00000534477 | LINC00958 | lincRNA |
| smORF_212 | 235 | 330 | 96 | ENST00000532541 | LINC00958 | lincRNA |
| smORF_213 | 290 | 574 | 285 | ENST00000606019 | RP11-682N22.1 | lincRNA |
| smORF_214 | 9 | 95 | 87 | ENST00000500538 | UBA6-AS1 | antisense |
| smORF_215 | 37 | 123 | 87 | ENST00000506606 | UBA6-AS1 | antisense |
| smORF_216 | 90 | 176 | 87 | ENST00000498917 | UBA6-AS1 | antisense |
| smORF_217 | 249 | 380 | 132 | ENST00000507869 | AC108142.1 | antisense |
| smORF_218 | 197 | 307 | 111 | ENST00000505537 | AC108142.1 | antisense |
| smORF_219 | 111 | 413 | 303 | ENST00000437764 | SERTAD4-AS1 | antisense |
| smORF_220 | 263 | 523 | 261 | ENST00000313807 | EIF3J-AS1 | lincRNA |
| smORF_221 | 70 | 195 | 126 | ENST00000550853 | LINC00094 | antisense |
| smORF_222 | 117 | 242 | 126 | ENST00000430633 | LINC00094 | antisense |
| smORF_223 | 61 | 186 | 126 | ENST00000432807 | LINC00094 | antisense |
| smORF_224 | 524 | 718 | 195 | ENST00000547492 | RP11-620J15.3 | lincRNA |
| smORF_225 | 355 | 549 | 195 | ENST00000546580 | RP11-620J15.3 | lincRNA |
| smORF_226 | 66 | 374 | 309 | ENST00000456816 | LINC00887 | lincRNA |
| smORF_227 | 165 | 473 | 309 | ENST00000429578 | LINC00887 | lincRNA |
| smORF_228 | 162 | 470 | 309 | ENST00000414120 | LINC00887 | lincRNA |
| smORF_229 | 143 | 274 | 132 | ENST00000397644 | LINC00887 | lincRNA |
| smORF_230 | 383 | 556 | 174 | ENST00000422761 | AC007040.7 | antisense |
| smORF_231 | 75 | 206 | 132 | ENST00000448869 | RP11-284F21.7 | antisense |
| smORF_232 | 145 | 333 | 189 | ENST00000444717 | RP4-614O4.11 | antisense |
| smORF_233 | 62 | 178 | 117 | ENST00000437488 | RP11-385J1.2 | antisense |
| smORF_234 | 59 | 175 | 117 | ENST00000451742 | RP11-385J1.2 | antisense |
| smORF_235 | 10 | 276 | 267 | ENST00000572856 | DLGAP1-AS2 | antisense |
| smORF_236 | 96 | 356 | 261 | ENST00000581725 | LINC00668 | lincRNA |
| smORF_237 | 132 | 314 | 183 | ENST00000527945 | LINC00958 | lincRNA |
| smORF_238 | 187 | 330 | 144 | ENST00000593237 | RP11-220I1.1 | lincRNA |
| smORF_239 | 154 | 297 | 144 | ENST00000592157 | RP11-220I1.1 | lincRNA |
| smORF_240 | 678 | 821 | 144 | ENST00000588403 | RP11-220I1.1 | lincRNA |
| smORF_241 | 1738 | 1881 | 144 | ENST00000588314 | RP11-220I1.1 | lincRNA |
| smORF_242 | 701 | 844 | 144 | ENST00000429493 | RP11-220I1.1 | lincRNA |
| smORF_243 | 595 | 726 | 132 | ENST00000572067 | CTC-479C5.10 | lincRNA |
| smORF_244 | 291 | 467 | 177 | ENST00000513560 | NNT-AS1 | antisense |
| smORF_245 | 84 | 182 | 99 | ENST00000501011 | RAB30-AS1 | lincRNA |
| smORF_246 | 103 | 201 | 99 | ENST00000526795 | RAB30-AS1 | lincRNA |
| smORF_247 | 271 | 369 | 99 | ENST00000530270 | RAB30-AS1 | lincRNA |
| smORF_248 | 28 | 126 | 99 | ENST00000534499 | RAB30-AS1 | lincRNA |
| smORF_249 | 67 | 165 | 99 | ENST00000533708 | RAB30-AS1 | lincRNA |
| smORF_250 | 84 | 182 | 99 | ENST00000527627 | RAB30-AS1 | lincRNA |
| smORF_251 | 116 | 328 | 213 | ENST00000581571 | LINC00668 | lincRNA |
| smORF_252 | 77 | 289 | 213 | ENST00000578497 | LINC00668 | lincRNA |
| smORF_253 | 116 | 328 | 213 | ENST00000583316 | LINC00668 | lincRNA |
| smORF_254 | 27 | 299 | 273 | ENST00000452148 | PCAT7 | antisense |
| smORF_255 | 96 | 308 | 213 | ENST00000539543 | RP11-14N7.2 | lincRNA |
| smORF_256 | 37 | 195 | 159 | ENST00000500741 | DYNLL1-AS1 | antisense |
| smORF_257 | 60 | 281 | 222 | ENST00000609932 | CECR7 | lincRNA |
| smORF_258 | 13 | 234 | 222 | ENST00000609596 | CECR7 | lincRNA |
| smORF_259 | 729 | 950 | 222 | ENST00000441006 | CECR7 | lincRNA |
| smORF_260 | 87 | 308 | 222 | ENST00000414401 | CECR7 | lincRNA |
| smORF_261 | 133 | 288 | 156 | ENST00000415330 | RP11-558F24.4 | antisense |
| smORF_262 | 6 | 134 | 129 | ENST00000553657 | RP11-349A22.5 | antisense |
| smORF_263 | 55 | 183 | 129 | ENST00000555037 | RP11-349A22.5 | antisense |
| smORF_264 | 143 | 382 | 240 | ENST00000520447 | SETD5-AS1 | antisense |
| smORF_265 | 54 | 254 | 201 | ENST00000551597 | RP11-349A22.5 | antisense |
| smORF_266 | 752 | 919 | 168 | ENST00000453396 | TOPORS-AS1 | antisense |
| smORF_267 | 237 | 404 | 168 | ENST00000450093 | TOPORS-AS1 | antisense |
| smORF_268 | 48 | 242 | 195 | ENST00000554309 | RP11-349A22.5 | antisense |
| smORF_269 | 358 | 564 | 207 | ENST00000562965 | LOXL1-AS1 | antisense |
| smORF_270 | 88 | 261 | 174 | ENST00000334298 | LINC00221 | lincRNA |
| smORF_271 | 107 | 280 | 174 | ENST00000449670 | LINC00221 | lincRNA |
| smORF_272 | 115 | 240 | 126 | ENST00000521653 | RP11-513O17.2 | lincRNA |
| smORF_273 | 168 | 266 | 99 | ENST00000577537 | RP11-344E13.3 | antisense |
| smORF_274 | 54 | 152 | 99 | ENST00000577860 | RP11-344E13.3 | antisense |
| smORF_275 | 58 | 156 | 99 | ENST00000582324 | RP11-344E13.3 | antisense |
| smORF_276 | 96 | 194 | 99 | ENST00000417232 | RP11-344E13.3 | antisense |
| smORF_277 | 104 | 193 | 90 | ENST00000439794 | RP11-344E13.3 | antisense |
| smORF_278 | 95 | 337 | 243 | ENST00000570080 | CTD-2270L9.4 | lincRNA |
| smORF_279 | 54 | 308 | 255 | ENST00000501173 | CTC-228N24.3 | lincRNA |
| smORF_280 | 151 | 405 | 255 | ENST00000606251 | CTC-228N24.3 | lincRNA |
| smORF_281 | 79 | 333 | 255 | ENST00000499346 | CTC-228N24.3 | lincRNA |
| smORF_282 | 53 | 277 | 225 | ENST00000421685 | ZNF674-AS1 | lincRNA |
| smORF_283 | 42 | 188 | 147 | ENST00000557660 | RP11-349A22.5 | antisense |
| smORF_284 | 80 | 325 | 246 | ENST00000413405 | PTCHD3P1 | antisense |
| smORF_285 | 61 | 204 | 144 | ENST00000414457 | PTCHD3P1 | antisense |
| smORF_286 | 62 | 199 | 138 | ENST00000608994 | PTCHD3P1 | antisense |
| smORF_287 | 47 | 163 | 117 | ENST00000427063 | PTCHD3P1 | antisense |
| smORF_288 | 62 | 106 | 45 | ENST00000537908 | PTCHD3P1 | antisense |
| smORF_289 | 39 | 83 | 45 | ENST00000446807 | PTCHD3P1 | antisense |
| smORF_290 | 28 | 72 | 45 | ENST00000445521 | PTCHD3P1 | antisense |
| smORF_291 | 17 | 61 | 45 | ENST00000438202 | PTCHD3P1 | antisense |
| smORF_292 | 33 | 77 | 45 | ENST00000423223 | PTCHD3P1 | antisense |
| smORF_293 | 62 | 106 | 45 | ENST00000609413 | PTCHD3P1 | antisense |
| smORF_294 | 51 | 161 | 111 | ENST00000583962 | RP11-344E13.3 | antisense |
| smORF_295 | 1515 | 1688 | 174 | ENST00000572067 | CTC-479C5.10 | lincRNA |
| smORF_296 | 12 | 257 | 246 | ENST00000424751 | LINC00839 | lincRNA |
| smORF_297 | 112 | 270 | 159 | ENST00000602414 | SNHG8 | lincRNA |
| smORF_298 | 66 | 146 | 81 | ENST00000577537 | RP11-344E13.3 | antisense |
| smORF_299 | 2 | 82 | 81 | ENST00000439794 | RP11-344E13.3 | antisense |
| smORF_300 | 39 | 74 | 36 | ENST00000417232 | RP11-344E13.3 | antisense |
| smORF_301 | 1 | 36 | 36 | ENST00000582324 | RP11-344E13.3 | antisense |
| smORF_302 | 55 | 258 | 204 | ENST00000417112 | RP11-554I8.2 | lincRNA |
| smORF_303 | 52 | 303 | 252 | ENST00000586949 | RP11-879F14.2 | lincRNA |
| smORF_304 | 93 | 203 | 111 | ENST00000602483 | SNHG8 | lincRNA |
| smORF_305 | 253 | 363 | 111 | ENST00000602819 | SNHG8 | lincRNA |
| smORF_306 | 108 | 218 | 111 | ENST00000602520 | SNHG8 | lincRNA |
| smORF_307 | 353 | 463 | 111 | ENST00000602573 | SNHG8 | lincRNA |
| smORF_308 | 453 | 563 | 111 | ENST00000602414 | SNHG8 | lincRNA |
| smORF_309 | 33 | 254 | 222 | ENST00000608034 | LINC00493 | lincRNA |
| smORF_310 | 17 | 250 | 234 | ENST00000473756 | LINC00973 | lincRNA |
| smORF_311 | 60 | 347 | 288 | ENST00000411646 | LINC00493 | lincRNA |
| smORF_312 | 564 | 641 | 78 | ENST00000371743 | ZFAS1 | antisense |
| smORF_313 | 122 | 199 | 78 | ENST00000428008 | ZFAS1 | antisense |
| smORF_314 | 122 | 199 | 78 | ENST00000326677 | ZFAS1 | antisense |
| smORF_315 | 564 | 641 | 78 | ENST00000450535 | ZFAS1 | antisense |
| smORF_316 | 564 | 641 | 78 | ENST00000441722 | ZFAS1 | antisense |
| smORF_317 | 564 | 641 | 78 | ENST00000417721 | ZFAS1 | antisense |
| smORF_318 | 60 | 344 | 285 | ENST00000435844 | LINC00493 | lincRNA |
| smORF_319 | 59 | 229 | 171 | ENST00000458653 | ZFAS1 | antisense |
| smORF_320 | 95 | 394 | 300 | ENST00000602845 | NCBP2-AS2 | lincRNA |
| smORF_321 | 58 | 228 | 171 | ENST00000426713 | LINC00116 | lincRNA |
| smORF_322 | 182 | 418 | 237 | ENST00000442982 | PSMD5-AS1 | antisense |
| smORF_323 | 264 | 476 | 213 | ENST00000414584 | LINC00152 | lincRNA |
| smORF_324 | 245 | 418 | 174 | ENST00000431558 | PRRT3-AS1 | antisense |
| smORF_325 | 171 | 437 | 267 | ENST00000609682 | ZBTB11-AS1 | antisense |
| smORF_326 | 216 | 482 | 267 | ENST00000536865 | ZBTB11-AS1 | antisense |
| smORF_327 | 24 | 176 | 153 | ENST00000601263 | AC006262.5 | lincRNA |
| smORF_328 | 5 | 160 | 156 | ENST00000500741 | DYNLL1-AS1 | antisense |
| smORF_329 | 190 | 303 | 114 | ENST00000600553 | FENDRR | lincRNA |
| smORF_330 | 379 | 507 | 129 | ENST00000591384 | CTD-2357A8.3 | lincRNA |
| smORF_331 | 264 | 392 | 129 | ENST00000586185 | CTD-2357A8.3 | lincRNA |
| smORF_332 | 724 | 867 | 144 | ENST00000591372 | LINC00665 | lincRNA |
| smORF_333 | 486 | 596 | 111 | ENST00000592404 | CTC-459F4.3 | lincRNA |
| smORF_334 | 404 | 514 | 111 | ENST00000587188 | CTC-459F4.3 | lincRNA |
| smORF_335 | 457 | 558 | 102 | ENST00000586784 | CTC-459F4.3 | lincRNA |
| smORF_336 | 75 | 350 | 276 | ENST00000362058 | CROCCP2 | lincRNA |
| smORF_337 | 458 | 604 | 147 | ENST00000560208 | CRNDE | lincRNA |
| smORF_338 | 529 | 651 | 123 | ENST00000547804 | LINC00941 | lincRNA |
| smORF_339 | 458 | 652 | 195 | ENST00000585917 | CTC-459F4.3 | lincRNA |
| smORF_340 | 29 | 103 | 75 | ENST00000543515 | RP11-392P7.6 | antisense |
| smORF_341 | 25 | 99 | 75 | ENST00000542078 | RP11-392P7.6 | antisense |
| smORF_342 | 191 | 310 | 120 | ENST00000412387 | AC007879.5 | antisense |
| smORF_343 | 132 | 362 | 231 | ENST00000504869 | THAP9-AS1 | antisense |
| smORF_344 | 352 | 597 | 246 | ENST00000501173 | CTC-228N24.3 | lincRNA |
| smORF_345 | 408 | 518 | 111 | ENST00000561058 | RP11-624L4.1 | antisense |
| smORF_346 | 70 | 258 | 189 | ENST00000595595 | RP11-464F9.20 | antisense |
| smORF_347 | 383 | 562 | 180 | ENST00000506584 | TMEM161B-AS1 | antisense |
| smORF_348 | 467 | 730 | 264 | ENST00000419746 | TTN-AS1 | antisense |
| smORF_349 | 374 | 637 | 264 | ENST00000585358 | TTN-AS1 | antisense |
| smORF_350 | 322 | 585 | 264 | ENST00000450692 | TTN-AS1 | antisense |
| smORF_351 | 363 | 593 | 231 | ENST00000589434 | TTN-AS1 | antisense |
| smORF_352 | 398 | 607 | 210 | ENST00000565797 | CTB-31O20.2 | lincRNA |
| smORF_353 | 278 | 481 | 204 | ENST00000588716 | TTN-AS1 | antisense |
| smORF_354 | 491 | 694 | 204 | ENST00000588244 | TTN-AS1 | antisense |
| smORF_355 | 286 | 471 | 186 | ENST00000592630 | TTN-AS1 | antisense |
| smORF_356 | 157 | 315 | 159 | ENST00000415561 | TTN-AS1 | antisense |
| smORF_357 | 455 | 613 | 159 | ENST00000585487 | TTN-AS1 | antisense |
| smORF_358 | 545 | 703 | 159 | ENST00000588804 | TTN-AS1 | antisense |
| smORF_359 | 578 | 736 | 159 | ENST00000589391 | TTN-AS1 | antisense |
| smORF_360 | 668 | 961 | 294 | ENST00000424094 | GNAS-AS1 | antisense |
| smORF_361 | 241 | 348 | 108 | ENST00000505541 | AC025171.1 | antisense |
| smORF_362 | 1462 | 1569 | 108 | ENST00000515108 | AC025171.1 | antisense |
| smORF_363 | 513 | 575 | 63 | ENST00000534477 | LINC00958 | lincRNA |
| smORF_364 | 576 | 638 | 63 | ENST00000532541 | LINC00958 | lincRNA |
| smORF_365 | 507 | 569 | 63 | ENST00000504230 | LINC00958 | lincRNA |
| smORF_366 | 481 | 543 | 63 | ENST00000529328 | LINC00958 | lincRNA |
| smORF_367 | 695 | 787 | 93 | ENST00000458468 | LINC00478 | lincRNA |
| smORF_368 | 450 | 542 | 93 | ENST00000453910 | LINC00478 | lincRNA |
| smORF_369 | 455 | 547 | 93 | ENST00000428669 | LINC00478 | lincRNA |
| smORF_370 | 564 | 656 | 93 | ENST00000400178 | LINC00478 | lincRNA |
| smORF_371 | 275 | 367 | 93 | ENST00000441820 | LINC00478 | lincRNA |
| smORF_372 | 464 | 556 | 93 | ENST00000602892 | LINC00478 | lincRNA |
| smORF_373 | 213 | 305 | 93 | ENST00000602280 | LINC00478 | lincRNA |
| smORF_374 | 363 | 455 | 93 | ENST00000602323 | LINC00478 | lincRNA |
| smORF_375 | 54 | 155 | 102 | ENST00000422847 | LINC00857 | lincRNA |
| smORF_376 | 232 | 360 | 129 | ENST00000438482 | AC108488.4 | lincRNA |
| smORF_377 | 972 | 1073 | 102 | ENST00000488745 | SNHG12 | antisense |
| smORF_378 | 346 | 447 | 102 | ENST00000461832 | SNHG12 | antisense |
| smORF_379 | 331 | 432 | 102 | ENST00000461448 | SNHG12 | antisense |
| smORF_380 | 78 | 179 | 102 | ENST00000475441 | SNHG12 | antisense |
| smORF_381 | 323 | 424 | 102 | ENST00000531126 | SNHG12 | antisense |
| smORF_382 | 322 | 378 | 57 | ENST00000445681 | GS1-124K5.4 | lincRNA |
| smORF_383 | 238 | 294 | 57 | ENST00000452565 | GS1-124K5.4 | lincRNA |
| smORF_384 | 168 | 263 | 96 | ENST00000584327 | SNHG15 | lincRNA |
| smORF_385 | 415 | 510 | 96 | ENST00000584686 | SNHG15 | lincRNA |
| smORF_386 | 322 | 567 | 246 | ENST00000412224 | KTN1-AS1 | antisense |
| smORF_387 | 76 | 336 | 261 | ENST00000581211 | RP11-527H14.3 | lincRNA |
| smORF_388 | 187 | 288 | 102 | ENST00000418850 | AC007879.5 | antisense |
| smORF_389 | 489 | 581 | 93 | ENST00000567644 | LOXL1-AS1 | antisense |
| smORF_390 | 251 | 589 | 339 | ENST00000580048 | LINC00909 | lincRNA |
| smORF_391 | 26 | 118 | 93 | ENST00000565689 | LOXL1-AS1 | antisense |
| smORF_392 | 1255 | 1515 | 261 | ENST00000451213 | RP11-421L21.3 | antisense |
| smORF_393 | 1015 | 1275 | 261 | ENST00000449473 | RP11-421L21.3 | antisense |
| smORF_394 | 429 | 653 | 225 | ENST00000560197 | RP11-624L4.1 | antisense |
| smORF_395 | 517 | 687 | 171 | ENST00000438436 | RNASEH1-AS1 | antisense |
| smORF_396 | 151 | 264 | 114 | ENST00000453910 | LINC00478 | lincRNA |
| smORF_397 | 237 | 350 | 114 | ENST00000435697 | LINC00478 | lincRNA |
| smORF_398 | 267 | 380 | 114 | ENST00000418813 | LINC00478 | lincRNA |
| smORF_399 | 601 | 855 | 255 | ENST00000512932 | THAP9-AS1 | antisense |
| smORF_400 | 1159 | 1413 | 255 | ENST00000504520 | THAP9-AS1 | antisense |
| smORF_401 | 1502 | 1756 | 255 | ENST00000504792 | THAP9-AS1 | antisense |
| smORF_402 | 558 | 710 | 153 | ENST00000606743 | XXbac-BPG181B23.7 | lincRNA |
| smORF_403 | 19 | 171 | 153 | ENST00000356684 | FLVCR1-AS1 | lincRNA |
| smORF_404 | 19 | 171 | 153 | ENST00000424044 | FLVCR1-AS1 | lincRNA |
| smORF_405 | 19 | 171 | 153 | ENST00000426161 | FLVCR1-AS1 | lincRNA |
| smORF_406 | 152 | 340 | 189 | ENST00000414798 | RP11-342M1.3 | antisense |
| smORF_407 | 2578 | 2769 | 192 | ENST00000588314 | RP11-220I1.1 | lincRNA |
| smORF_408 | 1541 | 1732 | 192 | ENST00000429493 | RP11-220I1.1 | lincRNA |
| smORF_409 | 408 | 542 | 135 | ENST00000521383 | RP11-318M2.2 | antisense |
| smORF_410 | 109 | 216 | 108 | ENST00000606998 | RP11-356I2.4 | antisense |
| smORF_411 | 272 | 343 | 72 | ENST00000409139 | LINC00152 | lincRNA |
| smORF_412 | 218 | 340 | 123 | ENST00000445565 | RP11-418J17.1 | antisense |
| smORF_413 | 321 | 407 | 87 | ENST00000504230 | LINC00958 | lincRNA |
| smORF_414 | 295 | 381 | 87 | ENST00000529328 | LINC00958 | lincRNA |
| smORF_415 | 327 | 413 | 87 | ENST00000534477 | LINC00958 | lincRNA |
| smORF_416 | 390 | 476 | 87 | ENST00000532541 | LINC00958 | lincRNA |
| smORF_417 | 504 | 590 | 87 | ENST00000526388 | LINC00958 | lincRNA |
| smORF_418 | 447 | 533 | 87 | ENST00000531402 | LINC00958 | lincRNA |
| smORF_419 | 1898 | 1957 | 60 | ENST00000429493 | RP11-220I1.1 | lincRNA |
| smORF_420 | 414 | 470 | 57 | ENST00000425533 | TOPORS-AS1 | antisense |
| smORF_421 | 201 | 257 | 57 | ENST00000458036 | TOPORS-AS1 | antisense |
| smORF_422 | 73 | 168 | 96 | ENST00000414554 | NIFK-AS1 | antisense |
| smORF_423 | 1705 | 1875 | 171 | ENST00000508913 | AC025171.1 | antisense |
| smORF_424 | 1695 | 1865 | 171 | ENST00000399543 | AC025171.1 | antisense |
| smORF_425 | 616 | 786 | 171 | ENST00000451894 | AC025171.1 | antisense |
| smORF_426 | 553 | 741 | 189 | ENST00000420471 | LINC00630 | lincRNA |
| smORF_427 | 305 | 472 | 168 | ENST00000431616 | LINC00630 | lincRNA |
| smORF_428 | 19 | 183 | 165 | ENST00000604760 | RP11-502I4.3 | lincRNA |
| smORF_429 | 420 | 518 | 99 | ENST00000446578 | Z83851.3 | lincRNA |
| smORF_430 | 1116 | 1205 | 90 | ENST00000332965 | Z83851.3 | lincRNA |
| smORF_431 | 126 | 212 | 87 | ENST00000602810 | ZSCAN16-AS1 | antisense |
| smORF_432 | 750 | 836 | 87 | ENST00000600652 | ZSCAN16-AS1 | antisense |
| smORF_433 | 1187 | 1249 | 63 | ENST00000564834 | LINC01003 | lincRNA |
| smORF_434 | 63 | 194 | 132 | ENST00000424523 | AC002454.1 | antisense |
| smORF_435 | 176 | 292 | 117 | ENST00000419668 | AC002454.1 | antisense |
| smORF_436 | 402 | 560 | 159 | ENST00000524003 | GS1-251I9.4 | antisense |
| smORF_437 | 90 | 290 | 201 | ENST00000426282 | CTA-217C2.1 | lincRNA |
| smORF_438 | 491 | 670 | 180 | ENST00000396588 | AC097662.2 | antisense |
| smORF_439 | 334 | 513 | 180 | ENST00000439598 | AC097662.2 | antisense |
| smORF_440 | 33 | 167 | 135 | ENST00000457853 | RP11-290F20.1 | lincRNA |
| smORF_441 | 329 | 442 | 114 | ENST00000592381 | RP11-1094M14.11 | lincRNA |
| smORF_442 | 373 | 561 | 189 | ENST00000609228 | MAPKAPK5-AS1 | lincRNA |
| smORF_443 | 1698 | 1874 | 177 | ENST00000456429 | MAPKAPK5-AS1 | lincRNA |
| smORF_444 | 293 | 469 | 177 | ENST00000590479 | MAPKAPK5-AS1 | lincRNA |
| smORF_445 | 6 | 272 | 267 | ENST00000558935 | NR2F2-AS1 | antisense |
| smORF_446 | 25 | 291 | 267 | ENST00000557863 | NR2F2-AS1 | antisense |
| smORF_447 | 192 | 293 | 102 | ENST00000502125 | NR2F2-AS1 | antisense |
| smORF_448 | 136 | 237 | 102 | ENST00000558929 | NR2F2-AS1 | antisense |
| smORF_449 | 158 | 259 | 102 | ENST00000560010 | NR2F2-AS1 | antisense |
| smORF_450 | 95 | 196 | 102 | ENST00000560800 | NR2F2-AS1 | antisense |
| smORF_451 | 188 | 289 | 102 | ENST00000560170 | NR2F2-AS1 | antisense |
| smORF_452 | 179 | 280 | 102 | ENST00000561344 | NR2F2-AS1 | antisense |
| smORF_453 | 87 | 182 | 96 | ENST00000499842 | RP11-1094H24.4 | lincRNA |
| smORF_454 | 243 | 383 | 141 | ENST00000602339 | LINC00478 | lincRNA |
| smORF_455 | 458 | 598 | 141 | ENST00000456342 | LINC00478 | lincRNA |
| smORF_456 | 434 | 574 | 141 | ENST00000445461 | LINC00478 | lincRNA |
| smORF_457 | 181 | 321 | 141 | ENST00000602620 | LINC00478 | lincRNA |
| smORF_458 | 403 | 543 | 141 | ENST00000418813 | LINC00478 | lincRNA |
| smORF_459 | 172 | 312 | 141 | ENST00000602901 | LINC00478 | lincRNA |
| smORF_460 | 179 | 427 | 249 | ENST00000437764 | SERTAD4-AS1 | antisense |
| smORF_461 | 31 | 282 | 252 | ENST00000431060 | RP11-66B24.7 | lincRNA |
| smORF_462 | 88 | 336 | 249 | ENST00000561231 | RP11-66B24.2 | lincRNA |
| smORF_463 | 87 | 335 | 249 | ENST00000559331 | RP11-66B24.2 | lincRNA |
| smORF_464 | 131 | 247 | 117 | ENST00000452322 | LINC00339 | lincRNA |
| smORF_465 | 340 | 456 | 117 | ENST00000420503 | LINC00339 | lincRNA |
| smORF_466 | 200 | 316 | 117 | ENST00000404210 | LINC00339 | lincRNA |
| smORF_467 | 200 | 316 | 117 | ENST00000416769 | LINC00339 | lincRNA |
| smORF_468 | 275 | 391 | 117 | ENST00000434233 | LINC00339 | lincRNA |
| smORF_469 | 172 | 261 | 90 | ENST00000413706 | AC091729.9 | antisense |
| smORF_470 | 284 | 481 | 198 | ENST00000424989 | LINC01137 | antisense |
| smORF_471 | 135 | 317 | 183 | ENST00000505028 | THAP9-AS1 | antisense |
| smORF_472 | 68 | 250 | 183 | ENST00000504718 | THAP9-AS1 | antisense |
| smORF_473 | 126 | 245 | 120 | ENST00000558687 | RP11-752G15.6 | antisense |
| smORF_474 | 379 | 435 | 57 | ENST00000456342 | LINC00478 | lincRNA |
| smORF_475 | 355 | 411 | 57 | ENST00000445461 | LINC00478 | lincRNA |
| smORF_476 | 371 | 427 | 57 | ENST00000419952 | LINC00478 | lincRNA |
| smORF_477 | 213 | 269 | 57 | ENST00000428669 | LINC00478 | lincRNA |
| smORF_478 | 164 | 220 | 57 | ENST00000602339 | LINC00478 | lincRNA |
| smORF_479 | 93 | 149 | 57 | ENST00000602901 | LINC00478 | lincRNA |
| smORF_480 | 2857 | 3114 | 258 | ENST00000421767 | LINC00174 | lincRNA |
| smORF_481 | 691 | 864 | 174 | ENST00000547804 | LINC00941 | lincRNA |
| smORF_482 | 448 | 621 | 174 | ENST00000551972 | LINC00941 | lincRNA |
| smORF_483 | 34 | 126 | 93 | ENST00000578280 | RP5-1028K7.2 | lincRNA |
| smORF_484 | 415 | 561 | 147 | ENST00000592381 | RP11-1094M14.11 | lincRNA |
| smORF_485 | 187 | 291 | 105 | ENST00000482677 | RP11-81N13.1 | lincRNA |
| smORF_486 | 248 | 352 | 105 | ENST00000464420 | RP11-81N13.1 | lincRNA |
| smORF_487 | 716 | 877 | 162 | ENST00000521369 | RP11-395G23.3 | lincRNA |
| smORF_488 | 58 | 111 | 54 | ENST00000529328 | LINC00958 | lincRNA |
| smORF_489 | 545 | 658 | 114 | ENST00000464242 | RP11-475N22.4 | antisense |
| smORF_490 | 25 | 99 | 75 | ENST00000442171 | RP11-452F19.3 | lincRNA |
| smORF_491 | 147 | 221 | 75 | ENST00000457636 | RP11-452F19.3 | lincRNA |
| smORF_492 | 395 | 478 | 84 | ENST00000436383 | LINC00707 | lincRNA |
| smORF_493 | 231 | 278 | 48 | ENST00000464242 | RP11-475N22.4 | antisense |
| smORF_494 | 574 | 621 | 48 | ENST00000468377 | RP11-475N22.4 | antisense |
| smORF_495 | 394 | 573 | 180 | ENST00000499346 | CTC-228N24.3 | lincRNA |
| smORF_496 | 87 | 266 | 180 | ENST00000514409 | CTC-228N24.3 | lincRNA |
| smORF_497 | 289 | 405 | 117 | ENST00000452728 | ENTPD1-AS1 | antisense |
| smORF_498 | 84 | 137 | 54 | ENST00000504230 | LINC00958 | lincRNA |
| smORF_499 | 24 | 305 | 282 | ENST00000506340 | CTC-338M12.4 | antisense |
| smORF_500 | 26 | 307 | 282 | ENST00000511331 | CTC-338M12.4 | antisense |
| smORF_501 | 620 | 682 | 63 | ENST00000422199 | LINC00162 | lincRNA |
| smORF_502 | 174 | 275 | 102 | ENST00000456217 | ARL5B-AS1 | lincRNA |
| smORF_503 | 202 | 465 | 264 | ENST00000597785 | RAB11B-AS1 | antisense |
| smORF_504 | 763 | 969 | 207 | ENST00000593581 | RAB11B-AS1 | antisense |
| smORF_505 | 117 | 227 | 111 | ENST00000515513 | RP11-267A15.1 | lincRNA |
| smORF_506 | 58 | 168 | 111 | ENST00000507361 | RP11-267A15.1 | lincRNA |
| smORF_507 | 323 | 547 | 225 | ENST00000500148 | CKMT2-AS1 | antisense |
| smORF_508 | 8 | 172 | 165 | ENST00000443623 | LINC-PINT | antisense |
| smORF_509 | 1787 | 1951 | 165 | ENST00000451786 | LINC-PINT | antisense |
| smORF_510 | 294 | 458 | 165 | ENST00000435523 | LINC-PINT | antisense |
| smORF_511 | 369 | 533 | 165 | ENST00000433079 | LINC-PINT | antisense |
| smORF_512 | 352 | 516 | 165 | ENST00000423414 | LINC-PINT | antisense |
| smORF_513 | 429 | 491 | 63 | ENST00000590677 | LINC00662 | lincRNA |
| smORF_514 | 65 | 223 | 159 | ENST00000585356 | LINC00665 | lincRNA |
| smORF_515 | 66 | 224 | 159 | ENST00000438368 | LINC00665 | lincRNA |
| smORF_516 | 7 | 165 | 159 | ENST00000591372 | LINC00665 | lincRNA |
| smORF_517 | 76 | 234 | 159 | ENST00000590622 | LINC00665 | lincRNA |
| smORF_518 | 241 | 276 | 36 | ENST00000414006 | ENTPD1-AS1 | antisense |
| smORF_519 | 148 | 183 | 36 | ENST00000452942 | ENTPD1-AS1 | antisense |
| smORF_520 | 149 | 184 | 36 | ENST00000427300 | ENTPD1-AS1 | antisense |
| smORF_521 | 156 | 191 | 36 | ENST00000449419 | ENTPD1-AS1 | antisense |
| smORF_522 | 31 | 108 | 78 | ENST00000544553 | RP11-783K16.5 | antisense |
| smORF_523 | 17 | 322 | 306 | ENST00000498917 | UBA6-AS1 | antisense |
| smORF_524 | 25 | 306 | 282 | ENST00000514109 | UBA6-AS1 | antisense |
| smORF_525 | 59 | 241 | 183 | ENST00000500538 | UBA6-AS1 | antisense |
| smORF_526 | 87 | 269 | 183 | ENST00000506606 | UBA6-AS1 | antisense |
| smORF_527 | 58 | 195 | 138 | ENST00000571639 | RP11-65J21.3 | lincRNA |
| smORF_528 | 116 | 253 | 138 | ENST00000575792 | RP11-65J21.3 | lincRNA |
| smORF_529 | 336 | 473 | 138 | ENST00000570945 | RP11-65J21.3 | lincRNA |
| smORF_530 | 555 | 713 | 159 | ENST00000544868 | MALAT1 | lincRNA |
| smORF_531 | 1838 | 1996 | 159 | ENST00000534336 | MALAT1 | lincRNA |
| smORF_532 | 247 | 336 | 90 | ENST00000438436 | RNASEH1-AS1 | antisense |
| smORF_533 | 161 | 250 | 90 | ENST00000426725 | RNASEH1-AS1 | antisense |
| smORF_534 | 102 | 284 | 183 | ENST00000597230 | CTD-3138B18.5 | antisense |
| smORF_535 | 22 | 132 | 111 | ENST00000513626 | LUCAT1 | lincRNA |
| smORF_536 | 3 | 113 | 111 | ENST00000511918 | LUCAT1 | lincRNA |
| smORF_537 | 180 | 293 | 114 | ENST00000500112 | CCAT1 | lincRNA |

Start = the start position of smORF in the transcript. End = the end position of smORF in the transcript. Length = the length of smORFs.

**Supplementary Table S5.** Translated smORFs validated by experimental methods.

| Name | Transcript | Length | LR | LDA | SVM | RF | Number |
| --- | --- | --- | --- | --- | --- | --- | --- |
| ZFAS1 | ENST00000458653 | 171 | 1 | 1 | 1 | 1 | 4 |
| RP11-879F14.2 | ENST00000586949 | 252 | 1 | 1 | 1 | 1 | 4 |
| SNHG8 | ENST00000602483 | 111 | 1 | 1 | 1 | 1 | 4 |
| RP4-614O4.11 | ENST00000444717 | 189 | 0 | 1 | 0 | 0 | 1 |
| RP11-554I8.2 | ENST00000417112 | 204 | 1 | 0 | 0 | 0 | 1 |

Length = the length of smORFs. LR = logistic regression. LDA = linear discriminant analysis. SVM = support vector machine. RF = random forest model. 1 = smORF predicted as a translated smORF by the classifier. 0 = smORF predicted as a non-coding smORF by the classifier. Number = sum of the prediction results of four classifiers based on U2OS RPF-Seq.

**Supplementary Table S6.** LncRNA-encoded micro-peptide fragments detected by MS.

| Symbol | Protein score | Peptide score | P-value | Peptide sequence |
| --- | --- | --- | --- | --- |
| LINC00909 | 14 | 13.54 | <0.05 | AGAVSPGGLSGISR |
| LINC00909 | 14 | 12.5 | <0.05 | SPLPLCSLPPGLWLLSLLALR |
| RP11-277P12.20 | 13 | 1.59 | <0.05 | MTDLLSPLPAILGPR |

Protein score, peptide score, and p-value were calculated by MaxQuant.

**Supplementary Table S7.** Significantly dysregulated translated lncRNAs in tumor expression profiles.

| GEO_ID | TYPE | ENSEMBL_ID | Mean_tumor | Mean_normal | FC | FDR |
| --- | --- | --- | --- | --- | --- | --- |
| GSE53137 | SC | ENST00000498917 | 26.05913 | 43.65995 | 0.596866 | 0.036458 |
| GSE53137 | SC | ENST00000580048  (LINC00909) | 695.5777 | 1677.496 | 0.414652 | 0.036458 |
| GSE53137 | SC | ENST00000603175 | 139.9447 | 77.69783 | 1.80114 | 0.036458 |
| GSE53137 | SC | ENST00000409139 | 67.53951 | 44.28177 | 1.525222 | 0.036458 |
| GSE53137 | SC | ENST00000437488 | 124.744 | 199.0015 | 0.62685 | 0.036458 |
| GSE53137 | SC | ENST00000431558 | 501.6399 | 1558.668 | 0.321839 | 0.036458 |
| GSE53137 | SC | ENST00000602845 | 267.3875 | 121.9638 | 2.192351 | 0.036458 |
| GSE53137 | SC | ENST00000500538 | 55.91238 | 87.48016 | 0.639144 | 0.036458 |
| GSE53137 | SC | ENST00000380970 | 327.5066 | 750.0435 | 0.43665 | 0.036458 |
| GSE53137 | SC | ENST00000431679 | 105.414 | 243.8699 | 0.432255 | 0.036458 |
| GSE53137 | SC | ENST00000430633 | 1494.748 | 2668.598 | 0.560125 | 0.036458 |
| GSE53137 | SC | ENST00000437322 | 52.94782 | 119.9129 | 0.441552 | 0.036458 |
| GSE53137 | SC | ENST00000414798 | 32.25791 | 51.84509 | 0.622198 | 0.036458 |
| GSE53137 | SC | ENST00000609803 | 545.6623 | 949.7281 | 0.574546 | 0.036458 |
| GSE53137 | SC | ENST00000554988 | 44.78692 | 157.7592 | 0.283894 | 0.036458 |
| GSE53137 | SC | ENST00000450804 | 10801.05 | 3619.642 | 2.984012 | 0.036458 |
| GSE53137 | SC | ENST00000563475 | 122.0222 | 227.7153 | 0.535854 | 0.036458 |
| GSE53137 | SC | ENST00000561521 | 154.1174 | 398.1398 | 0.387094 | 0.036458 |
| GSE53137 | SC | ENST00000572856 | 278.5154 | 144.3262 | 1.929763 | 0.1 |
| GSE58043 | HCC | ENST00000429940 | 15.98714 | 7.491429 | 2.134058 | 0.055339 |
| GSE58043 | HCC | ENST00000468186 | 103.6714 | 22.22 | 4.665681 | 0.055339 |
| GSE58043 | HCC | ENST00000456429 | 526 | 188.2857 | 2.793627 | 0.055339 |
| GSE58043 | HCC | ENST00000437322 | 54.62857 | 14.72 | 3.71118 | 0.055339 |
| GSE58043 | HCC | ENST00000561521 | 149.6571 | 66.42857 | 2.252903 | 0.055339 |
| GSE58043 | HCC | ENST00000438482 | 130.2143 | 71.54286 | 1.820088 | 0.055339 |
| GSE58043 | HCC | ENST00000427063 | 409.2857 | 148.4143 | 2.757725 | 0.055339 |
| GSE58043 | HCC | ENST00000511918 | 357.0286 | 54.77143 | 6.518519 | 0.055339 |
| GSE58043 | HCC | ENST00000411646 | 19128.57 | 9630 | 1.986352 | 0.055339 |
| GSE58043 | HCC | ENST00000409139 | 78.87143 | 16.89571 | 4.668132 | 0.055339 |
| GSE58043 | HCC | ENST00000539543 | 543.0286 | 56.77143 | 9.565174 | 0.055339 |
| GSE58043 | HCC | ENST00000522525 | 62.54286 | 31.92857 | 1.958837 | 0.055339 |
| GSE58043 | HCC | ENST00000438436 | 22.9 | 9.88 | 2.317814 | 0.055339 |
| GSE58043 | HCC | ENST00000426713 | 6320 | 3084.286 | 2.049097 | 0.055339 |
| GSE58043 | HCC | ENST00000399543 | 233.4714 | 56.74286 | 4.114552 | 0.055339 |
| GSE58043 | HCC | ENST00000524003 | 135.5571 | 31.92857 | 4.245638 | 0.055339 |
| GSE58043 | HCC | ENST00000582106 | 73.07143 | 34.07143 | 2.144654 | 0.055339 |
| GSE58043 | HCC | ENST00000428029 | 178.8429 | 64.9 | 2.755668 | 0.055339 |
| GSE58043 | HCC | ENST00000563261 | 153.4 | 49.27429 | 3.113186 | 0.055339 |
| GSE58043 | HCC | ENST00000507044 | 571.5714 | 149.9429 | 3.811928 | 0.055339 |
| GSE58043 | HCC | ENST00000416970 | 1228.857 | 457.2857 | 2.687285 | 0.055339 |
| GSE58043 | HCC | ENST00000458653  (ZFAS1) | 4887.143 | 1208.571 | 4.043735 | 0.055339 |
| GSE58043 | HCC | ENST00000428207 | 694 | 361.2857 | 1.920917 | 0.055339 |
| GSE58043 | HCC | ENST00000434627 | 13.00857 | 27.10857 | 0.479869 | 0.055339 |
| GSE58043 | HCC | ENST00000501177 | 81.47143 | 18.51 | 4.401482 | 0.080492 |
| GSE58043 | HCC | ENST00000563475 | 240.2571 | 101.3143 | 2.371404 | 0.080492 |
| GSE58043 | HCC | ENST00000421685 | 400.2857 | 81.18571 | 4.930494 | 0.080492 |
| GSE58043 | HCC | ENST00000553985 | 41.68571 | 21.55714 | 1.933731 | 0.080492 |
| GSE58043 | HCC | ENST00000609803 | 184.3857 | 84.14286 | 2.191341 | 0.080492 |
| GSE58043 | HCC | ENST00000452399 | 1134 | 168.7714 | 6.719147 | 0.080492 |
| GSE58043 | HCC | ENST00000501122 | 233.0929 | 501.2857 | 0.46499 | 0.080492 |
| GSE58043 | HCC | ENST00000430633 | 759.8571 | 479.5714 | 1.58445 | 0.080492 |
| GSE58043 | HCC | ENST00000441006 | 29.47429 | 5.42 | 5.43806 | 0.080492 |
| GSE58043 | HCC | ENST00000356684 | 41.72714 | 8.018571 | 5.203813 | 0.1 |
| GSE58043 | HCC | ENST00000582008 | 109.7857 | 52.28571 | 2.099727 | 0.1 |
| GSE58043 | HCC | ENST00000456816 | 68.83714 | 9.465714 | 7.272261 | 0.1 |
| GSE58043 | HCC | ENST00000511331 | 153.1429 | 83.98571 | 1.823439 | 0.1 |

Mean_tumor and Mean_normal represents the mean gene expression levels in tumor and normal tissues, respectively. HCC, SC and GBM means hepatocellular carcinoma, gastric cancer and glioblastoma, respectively. FC means fold change of gene expression level. FDR means false discovery rate corrected by Bonferroni method.

**Supplementary Table S8.** Differentially expressed genes following ZFAS1 overexpression.

| Gene symbol | logFC | FDR | PCDH | ZFAS1 | Si-GFP | Si-ZFAS1 |
| --- | --- | --- | --- | --- | --- | --- |
| NDUFA6 | -0.87 | 1.7E-04 | 34.9 | 19.4 | 26.0 | 32.2 |
| NDUFA7 | -0.99 | 1.0E-04 | 27.6 | 14.8 | 8.9 | 29.0 |
| NDUFB4 | -0.61 | 2.6E-02 | 135.0 | 108.6 | 104.2 | 187.6 |
| NDUFB11 | -0.77 | 8.1E-04 | 67.7 | 41.0 | 46.4 | 72.7 |
| RPS19 | -1.57 | 5.6E-17 | 909.9 | 291.7 | 407.1 | 1054.9 |
| ROMO1 | -1.54 | 1.2E-16 | 244.6 | 87.1 | 85.6 | 192.3 |
| RPS27 | -1.38 | 8.3E-14 | 901.7 | 328.6 | 299.0 | 724.8 |
| ATP5I | -1.15 | 3.6E-07 | 74.2 | 30.1 | 44.1 | 79.0 |
| RPS20 | -1.11 | 4.6E-08 | 907.5 | 425.4 | 487.6 | 770.4 |
| HIST1H1C | -1.09 | 2.0E-05 | 21.2 | 10.6 | 5.9 | 18.0 |
| RPS15A | -1.08 | 4.8E-08 | 626.9 | 391.3 | 392.5 | 489.0 |
| RPL27 | -1.08 | 1.1E-07 | 1127.0 | 533.7 | 878.3 | 978.4 |
| RPL26L1 | -1.03 | 1.8E-06 | 51.7 | 29.0 | 28.1 | 39.5 |
| TMSB10 | -1.02 | 2.4E-06 | 5200.5 | 2614.7 | 1854.3 | 3549.9 |
| BOLA3 | -1.01 | 1.0E-04 | 24.9 | 12.9 | 10.8 | 21.2 |
| RPL22L1 | -1.01 | 1.5E-06 | 126.9 | 56.9 | 51.6 | 124.8 |
| RPL32 | -0.99 | 1.5E-06 | 707.6 | 347.1 | 343.6 | 583.0 |
| RPL35 | -0.99 | 2.5E-06 | 1489.5 | 771.1 | 771.8 | 1569.3 |
| CCDC167 | -0.97 | 1.2E-03 | 16.9 | 8.9 | 14.6 | 14.9 |
| RPS26 | -0.97 | 2.0E-06 | 423.5 | 238.7 | 227.8 | 348.3 |
| MRPL54 | -0.96 | 1.4E-03 | 18.5 | 9.9 | 12.0 | 26.7 |
| RPS28 | -0.95 | 3.9E-06 | 320.1 | 163.2 | 150.6 | 277.4 |
| UBL5 | -0.94 | 6.9E-06 | 107.8 | 57.9 | 67.2 | 132.7 |
| RPL39 | -0.94 | 5.7E-05 | 615.4 | 348.6 | 429.9 | 438.3 |
| RPL30 | -0.94 | 6.6E-06 | 680.4 | 342.3 | 398.6 | 616.4 |
| RPS12 | -0.91 | 1.8E-05 | 704.1 | 400.3 | 331.2 | 588.1 |
| RPS15 | -0.85 | 8.2E-05 | 845.1 | 474.8 | 396.9 | 839.5 |
| SOD1 | -0.85 | 5.7E-05 | 144.1 | 83.0 | 91.9 | 137.3 |
| UQCRQ | -0.84 | 1.1E-04 | 166.7 | 91.0 | 113.3 | 179.6 |
| FAU | -0.84 | 1.0E-04 | 331.4 | 232.8 | 281.0 | 382.3 |
| RPS8 | -0.83 | 2.9E-04 | 1102.1 | 663.8 | 612.5 | 1043.4 |
| RPL9 | -0.81 | 2.3E-04 | 570.3 | 375.6 | 468.3 | 517.0 |
| DDT | -0.81 | 3.8E-04 | 129.0 | 80.5 | 54.5 | 96.2 |
| DRAP1 | -0.79 | 3.8E-04 | 452.9 | 235.9 | 246.2 | 409.8 |
| NOP10 | -0.79 | 5.5E-04 | 95.8 | 59.6 | 55.2 | 95.8 |
| POLR2I | -0.79 | 5.8E-04 | 113.7 | 68.0 | 57.8 | 113.5 |
| PHPT1 | -0.77 | 6.8E-04 | 90.4 | 53.0 | 43.2 | 87.8 |
| RPL24 | -0.75 | 1.1E-03 | 466.4 | 272.8 | 298.3 | 533.6 |
| RPS11 | -0.75 | 2.1E-03 | 1231.3 | 684.4 | 758.9 | 1266.6 |
| ATOX1 | -0.74 | 4.3E-03 | 54.0 | 32.2 | 44.8 | 65.1 |
| POP7 | -0.74 | 3.2E-03 | 39.2 | 28.4 | 29.2 | 49.6 |
| SLIRP | -0.72 | 2.9E-03 | 121.8 | 75.1 | 117.5 | 120.2 |
| NHP2 | -0.72 | 2.3E-03 | 94.4 | 60.9 | 84.5 | 107.4 |
| RPS16 | -0.72 | 3.1E-03 | 1039.3 | 657.3 | 724.3 | 938.6 |
| RPS3A | -0.72 | 2.1E-03 | 469.9 | 302.3 | 270.6 | 414.2 |
| USE1 | -0.72 | 8.1E-03 | 30.0 | 19.7 | 20.4 | 24.3 |
| RPL12 | -0.72 | 3.5E-03 | 814.7 | 529.5 | 469.5 | 730.9 |
| ATPIF1 | -0.72 | 3.5E-03 | 81.7 | 45.7 | 44.5 | 76.6 |
| COX14 | -0.70 | 6.9E-03 | 62.2 | 39.2 | 39.3 | 41.8 |
| RPL37A | -0.70 | 5.9E-03 | 1554.6 | 926.3 | 787.0 | 1529.9 |
| RPL10 | -0.70 | 9.1E-03 | 2039.2 | 1350.7 | 1102.6 | 1619.4 |
| C8orf59 | -0.70 | 1.7E-02 | 40.5 | 23.4 | 20.5 | 38.6 |
| RPS7 | -0.69 | 6.3E-03 | 842.9 | 545.8 | 461.8 | 797.4 |
| GADD45GIP1 | -0.69 | 1.2E-02 | 37.3 | 23.3 | 27.3 | 42.3 |
| RPL41 | -0.69 | 4.3E-03 | 756.6 | 470.5 | 468.2 | 676.2 |
| RPL14 | -0.68 | 5.9E-03 | 550.2 | 353.4 | 388.1 | 545.6 |
| RBP7 | -0.67 | 1.6E-02 | 44.1 | 28.9 | 24.9 | 31.7 |
| PPP1R14B | -0.66 | 7.7E-03 | 214.6 | 133.4 | 161.8 | 219.3 |
| MRPL51 | -0.66 | 1.1E-02 | 102.5 | 67.8 | 72.5 | 99.3 |
| IFI27L2 | -0.65 | 4.7E-02 | 36.0 | 23.1 | 21.2 | 41.4 |
| RPS24 | -0.65 | 1.6E-02 | 1365.8 | 884.0 | 946.5 | 1594.0 |
| RPL31 | -0.65 | 1.6E-02 | 1769.0 | 1092.0 | 1110.0 | 1465.2 |
| MYEOV2 | -0.65 | 2.9E-02 | 65.5 | 44.1 | 52.1 | 80.3 |
| RPL7A | -0.64 | 2.5E-02 | 1627.6 | 1095.6 | 1262.9 | 1435.5 |
| RPL23A | -0.64 | 1.8E-02 | 1096.9 | 710.2 | 741.3 | 1151.5 |
| PSMC1 | -0.64 | 1.7E-02 | 52.2 | 38.9 | 36.6 | 50.5 |
| COA3 | -0.64 | 1.6E-02 | 86.2 | 58.8 | 70.5 | 87.1 |
| UQCR11 | -0.63 | 2.4E-02 | 56.9 | 33.1 | 33.2 | 60.6 |
| C17orf89 | -0.63 | 3.6E-02 | 51.2 | 36.5 | 45.1 | 48.4 |
| LSM10 | -0.63 | 3.3E-02 | 32.1 | 20.9 | 24.5 | 31.3 |
| RPL19 | -0.63 | 2.9E-02 | 1755.8 | 1207.1 | 1342.4 | 1965.4 |
| COX6C | -0.63 | 2.3E-02 | 100.3 | 64.2 | 62.5 | 104.2 |
| STARD3NL | -0.62 | 2.3E-02 | 41.4 | 26.0 | 29.2 | 31.5 |
| NME1 | -0.62 | 2.0E-02 | 183.2 | 120.8 | 136.3 | 207.2 |
| MRPL41 | -0.62 | 2.6E-02 | 41.0 | 27.3 | 32.2 | 39.0 |
| ATP5G2 | -0.62 | 2.0E-02 | 170.4 | 118.5 | 102.5 | 178.2 |
| MRPL40 | -0.62 | 2.6E-02 | 44.2 | 29.9 | 30.4 | 41.1 |
| MRPL33 | -0.61 | 3.2E-02 | 100.2 | 58.4 | 74.3 | 110.2 |
| PRKCDBP | -0.61 | 3.7E-02 | 27.9 | 18.1 | 17.8 | 29.3 |
| TMA7 | -0.61 | 2.9E-02 | 133.7 | 94.1 | 72.7 | 130.9 |
| LSM7 | -0.61 | 3.6E-02 | 75.3 | 45.8 | 51.4 | 89.4 |
| RPL29 | -0.60 | 3.3E-02 | 815.3 | 620.9 | 648.6 | 935.9 |
| RPL35A | -0.60 | 3.9E-02 | 1060.2 | 735.2 | 818.8 | 1303.6 |
| RPL37 | -0.59 | 3.7E-02 | 559.7 | 294.9 | 254.6 | 566.3 |
| MRPL52 | -0.59 | 4.8E-02 | 74.9 | 50.0 | 80.0 | 97.9 |
| EIF4EBP1 | -0.58 | 4.8E-02 | 81.0 | 56.2 | 53.2 | 72.3 |
| HELZ2 | 0.59 | 3.7E-02 | 13.8 | 20.5 | 15.8 | 15.7 |
| RTN2 | 1.04 | 4.0E-06 | 7.0 | 19.6 | 24.8 | 13.7 |
| MT-ND6 | 1.34 | 6.7E-11 | 23.3 | 67.5 | 39.0 | 28.0 |
| BCMO1 | 1.37 | 3.5E-03 | 1.8 | 9.9 | 11.0 | 4.0 |

LogFC means provided the fold change of gene expression of PCDH-ZFAS1-ORF overexpression comparing with PCDH. FDR. PCDH and si-GFP represents the mean gene expression of ZFAS1 overexpression and RNA-inference control. Si-GFP means the mean gene expression profiles of si control. Si means the mean gene expression profiles of two si.

**Supplementary Figure**


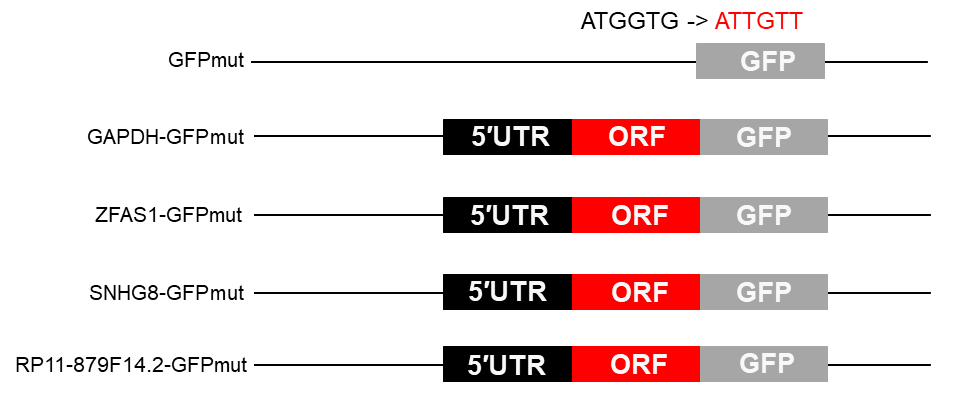


**Supplementary Figure S1. Construct structure of experimentally validated translated smORFs and their controls.** GFPmut means GFP start codon ATGGTG was mutated to ATTGTT.

.


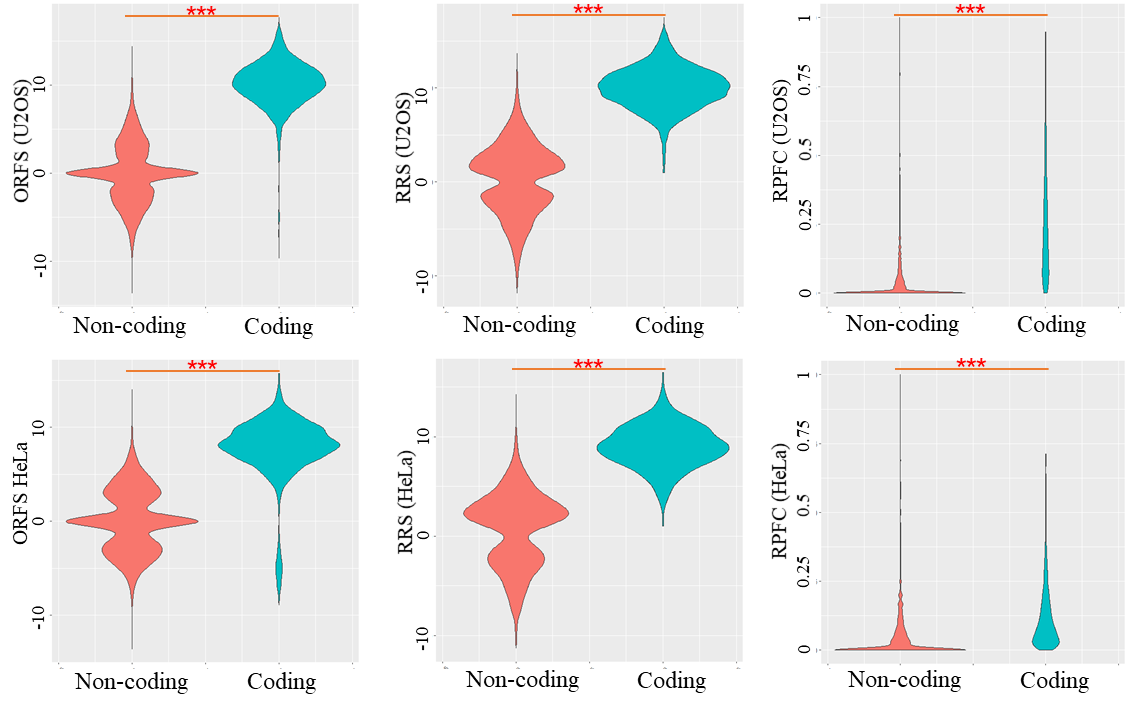


**Supplementary Figur**e **S2. The comparison of three ribosome features between positive and negative ORFs in the training cohort.** ORFS, RRS and RPFC means ORF score, ribosome release score and RPF coverage, respectively. *** means the *P-value* of Student's t-test < 0.0001.


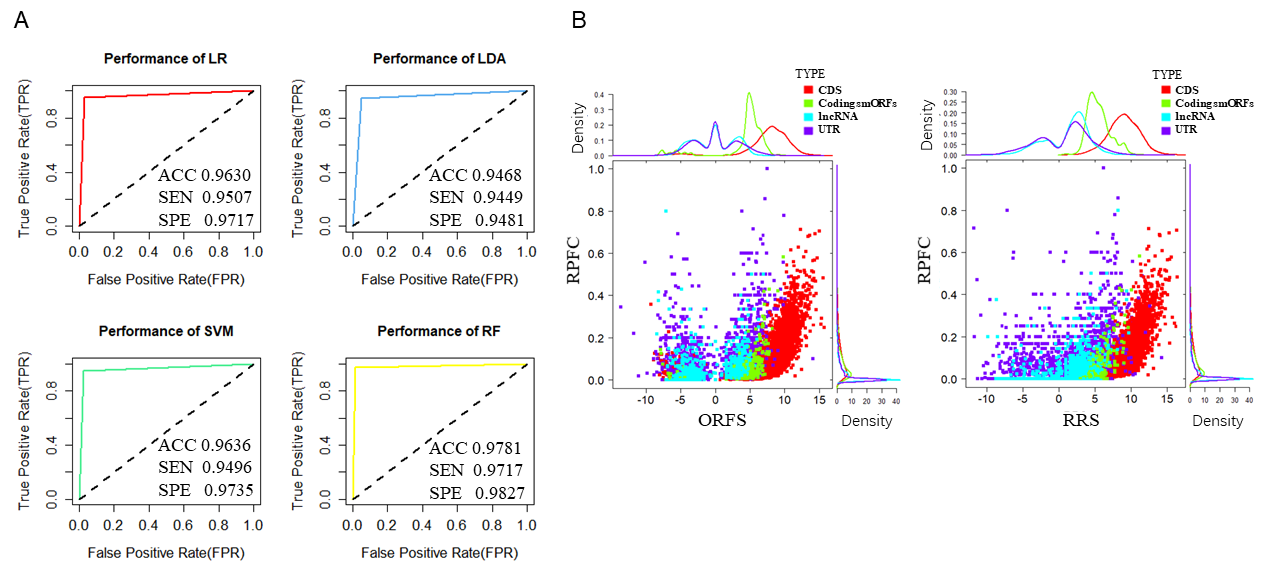


**Supplementary Figure S3. Features of predicted translated smORFs based on HeLa RPF-Seq.** (A) The performance of the four classifiers based on logistic regression (LR), linear discriminant analysis (LDA), support vector machine (SVM), and random forest models (RF). (B) Ribosome features of different ORFs. The ribosome release score and ORF score values of putative translated smORFs were similar, and were higher than ORFs derived from untranslated regions (UTRs) and long non-coding RNAs (lncRNAs) but lower than annotated ORFs of protein-coding genes. The ribosome-protected mRNA fragment coverage scores of translated smORFs and protein-coding genes were similar, but their distributions differed substantially from UTRs and lncRNAs. ACC, SEN, SPE means accuracy, sensitivity, and specificity, respectively. And RPFC, ORFS, RRS means ribosome-protected mRNA fragments coverage, ORF score, ribosome release score.

**
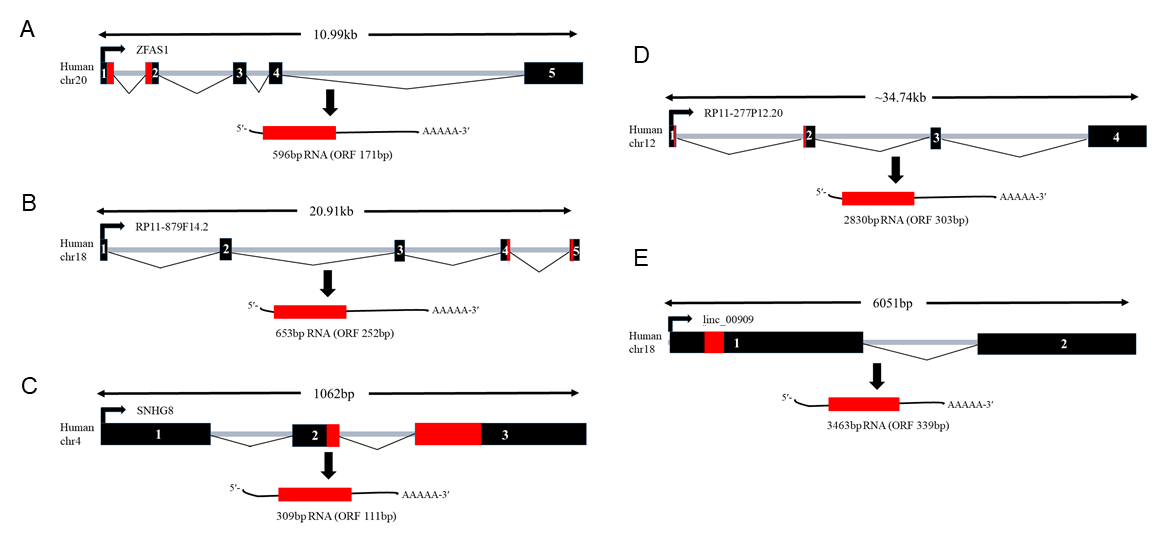
**

**Supplementary Figure S4. Gene structure of five experimentally validated translated smORFs.** (A) ZFAS1, the transcripts of ZFAS1 was 596 nt and the translated smORFs was 171 nt. (B) RP11-879F14.2, the transcripts of RP11-879F14.2 was 653 nt and the translated smORFs was 252 nt. (C) SNHG8, the transcripts of SNHG8 was 309 nt and the translated smORFs was 111 nt. (D) RP11-277P12.20, the transcripts of RP11-277P12.20 was 2830 nt and the translated smORFs was 303 nt. (E) linc_00909, the transcripts of linc_00909 was 3463 nt and the translated smORFs was 339 nt.


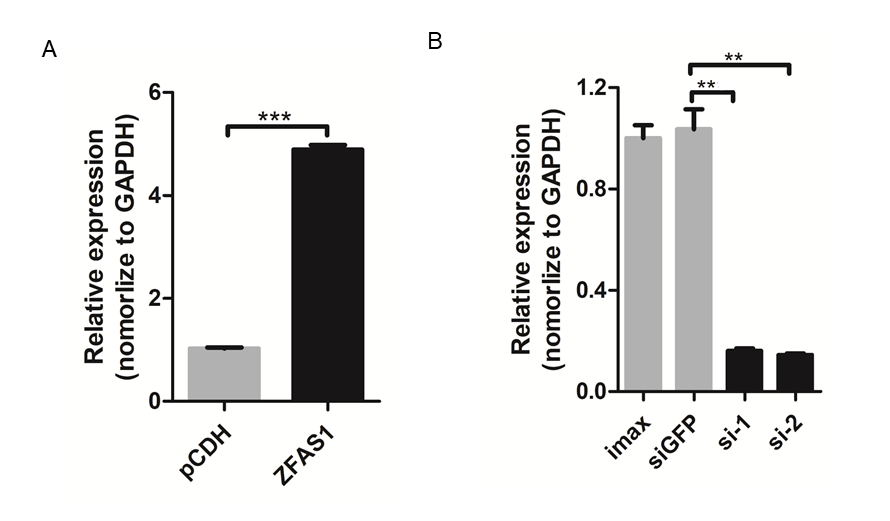


**Supplementary Figure S5. ZFAS1 expression levels after SK-Hep1 transfected with expression plasmid or siRNAs.** (A) After SK-Hep1 cells transfected with ZFAS1 expression plasmid, ZFAS1 expression level was significantly increased comparing with pCDH control (pCDH: *P-value*=0.253 and ZFAS1: *P-value*=0.274, Kolmogorov-Smimov test [K-S test]; *P-value=*0.1083*,* Brown-Forsythe test; *P-value*=6e-04, paired Student’s t-test, n=3). (B) After SK-Hep1 cells transfected with two independent siRNAs, ZFAS1 expression level was significantly decreased comparing with imax and siGFP control (imax: *P-value*=0.415, siGFP: *P-value*=0.948, si-1: *P-value*=0.168 and si-2: *P-value*=0.281, K-S test; Si-GFP vs si-1: *P-value=*3.09e-02, Si-GFP vs si-1: *P-value=*4e-04, Brown-Forsythe test; Si-GFP vs si-1: *P-value=*8.1e-03, Si-GFP vs si-1: *P-value=*7.8e-03, Welch's t-test, n=3).


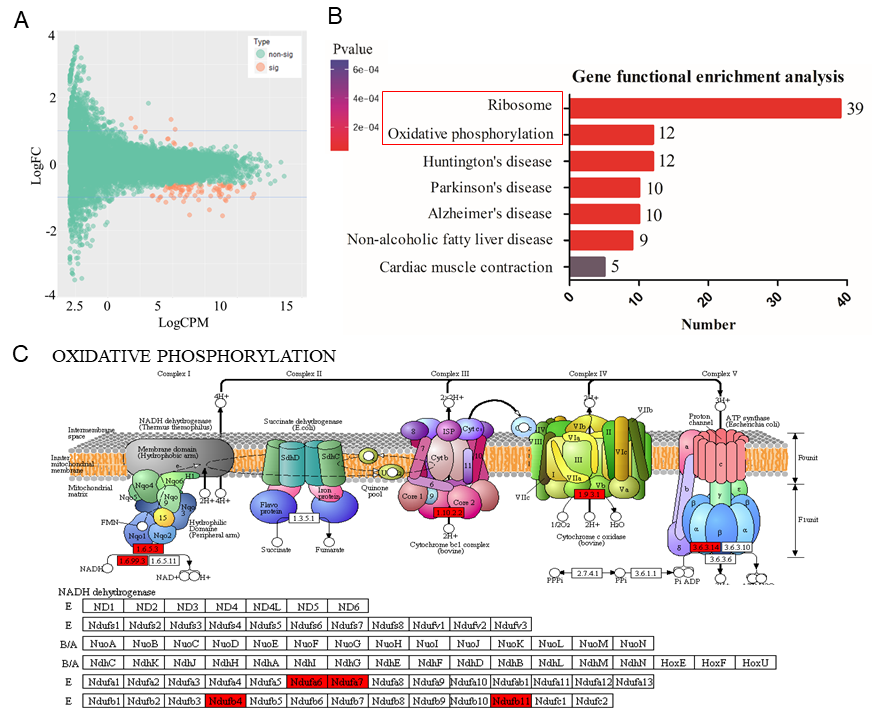


**Supplementary Figure S6.** The differentially expressed genes and enriched pathways. (A) The differentially expressed genes after SK-Hep1 cells transfected with expression plasmid. Totally, 101 and 10 significantly down-regulated and up-regulated genes were identified, respectively. (B) The pathway enrichment analysis of the significantly differentially expressed genes. (C) Differentially expressed genes in the oxidative phosphorylation.

**
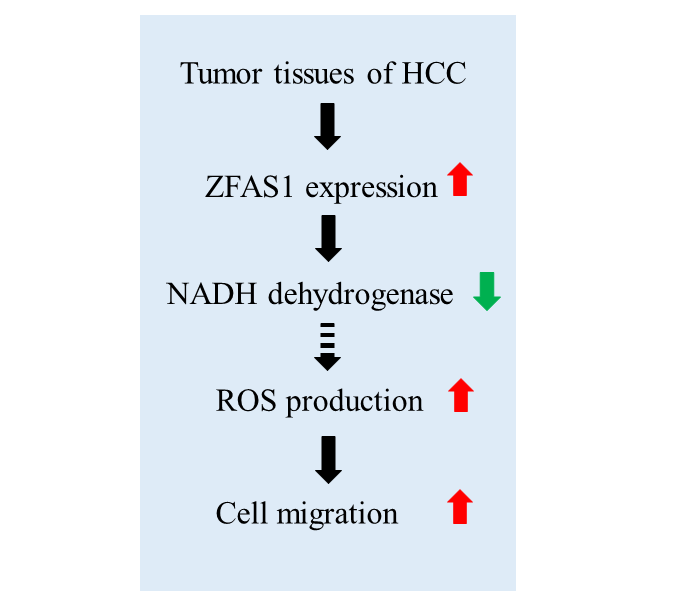
**

**Supplementary Figure S7.** The hypothesis of ZFAS1 mechanisms for regulating cancer cell migration. In the public datasets and 32 pairs HCC tissues, ZFAS1 expression levels was significantly increased in tumor tissues. After SK-Hep1 cells transfected with ZFAS1 expression plasmid, cell migration and ROS production were elevated and NADH dehydrogenase expression was down-regulated. Previous studies have revealed that down-regulated NADH dehydrogenase promotes cell migration by increasing intracellular ROS production. Furthermore, ROS has proven to serve as signaling molecules to regulate cell migration. Therefore, we supposed that up-regulated ZFAS1 promoted cancer cell migration by elevating cellular ROS production through repressing the expression of NADH dehydrogenase, including NDUFA6, NDUFB4, and NDUFB11.


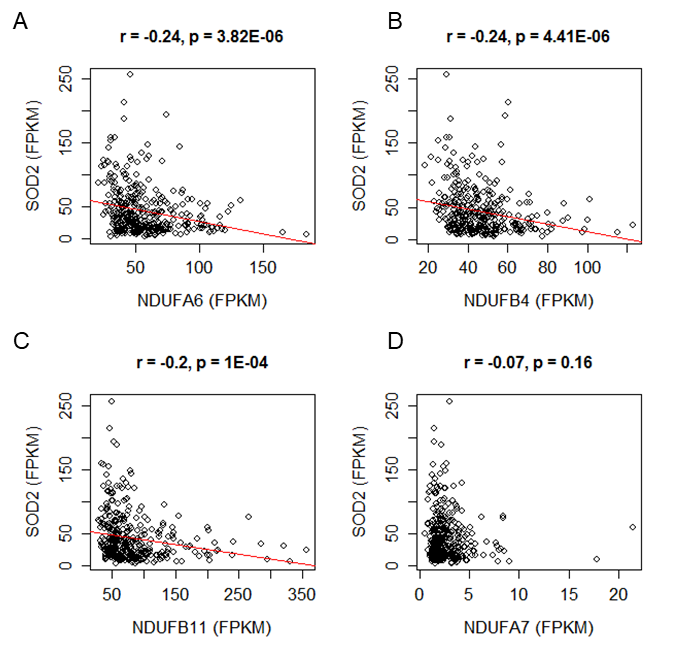


**Supplementary Figure S8.** Gene expression correlation between SOD2 and NADH dehydrogenase in HCC. (A) NDUFA6 was significantly negatively correlated with SOD2 (r=-0.24, *P-value*=3.82e-06). (B) NDUFB4 was significantly negatively correlated with SOD2 (r=-0.24, *P-value*=4.41e-06). (C) NDUFB11 was significantly negatively correlated with SOD2 (r=-0.2, *P-value*=1e-04). (D) NDUFA7 was not correlated with SOD2.
